# Efficient aqueous solubilization of methylxanthines *via* complexation with natural polyphenolate salts

**DOI:** 10.1039/d5ra09568a

**Published:** 2026-04-23

**Authors:** E. Hamonou, S. Antonczak, I. G. Shenderovich, D. Touraud, W. Kunz, N. Papaïconomou

**Affiliations:** a Université Côte d'Azur, CNRS, Institut de Chimie de Nice UMR 7272 Nice France Nicolas.PAPAICONOMOU@univ-cotedazur.fr; b Universität Regensburg, Institute of Physical and Theoretical Chemistry D-93040 Regensburg Germany

## Abstract

Caffeine, theophylline and theobromine are methylxanthines, a class of alkaloids present in coffee, tea and cocoa and therefore widely consumed all around the world. They exhibit several biological properties making them useful ingredients in cosmetics, as food additives or in pharmaceutical formulations. However, their solubility in water is limited. Extraction and purification often require the use of petrochemical volatile organic solvents. In the present paper, the solubility in water of caffeine, theophylline and theobromine in the presence of various natural solubilizing agents is reported. Niacinamide, as well as sodium or cholinium levulinate, salicylate, ferulate or caffeate were used to enhance the solubility of these methylxanthines in water. For example, solubility of theobromine was found to increase by a factor of 300 in aqueous solutions of ferulate salt. Solubilization mechanisms were investigated experimentally using ^1^H NMR and 2D ROESY-NMR and theoretically using molecular dynamics simulations. Both studies point towards the presence of π-stacking-type interactions between methylxanthines and solubilizing compounds. Benzene-based moieties exhibit stronger interactions and π-stacking with caffeine than pyridine-based moieties, such as nicotinic acid and niacinamide. These compounds can therefore be considered as complexing agents for methylxanthines rather than hydrotropes. Importantly, the linear dependence of solubility on solubilizing agent concentration indicates that these complexes maintain their solubilized state upon dilution, thereby enabling complete miscibility in water without precipitation.

## Abstract 2

Polyphenolate salts enable highly efficient aqueous solubilization of caffeine. The solubility enhancement originates from π–π stacking-driven complexation, offering a green alternative to conventional extraction media.

## Graphical abstract

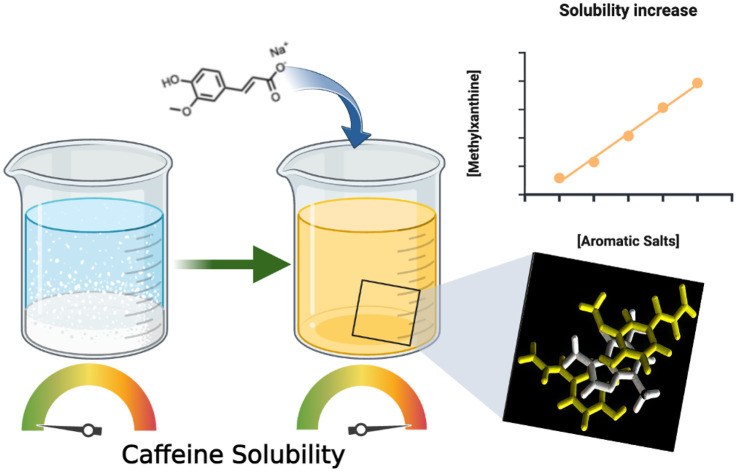

## Introduction

Caffeine, theophylline and theobromine belong to the methylxanthine family, a sub-class of alkaloids, which are secondary metabolites found in three of the most consumed drinks in the world: coffee, tea, and chocolate.^1,2^ For example caffeine consumption is estimated to be of 5.5 million tons per year in the world, incdluding coffee consumption, energy drinks, cosmetics, sport complements *etc.*^3–5^ These molecules also present some interesting properties for health.^6,7^ Caffeine is known to be beneficial against a large range of diseases: diabetes, Alzheimer's disease, heart diseases, Parkinson's disease and presents cosmetical benefits.^8–14^ Another important activity of caffeine is its use as a natural repellent.^15^ Theophylline and theobromine have also been reported to be effective in several fields of application. Theophylline has been studied for its anti-ageing effects, anti-inflammatory effects, anti-cancer activities, whereas theobromine exerts other interesting activities such as anti-tumoral ones. It is also a cardiovascular protector and used as a cosmetic agent.^16–20^

However, these three compounds, and by extension xanthine's group molecules, are poorly soluble in water. This can be explained when looking at their structure. Caffeine, theophylline and theobromine share a common structure that is one imidazole, and one pyrimidine ring bound together. These large, delocalized π-systems lead to hydrophobic interactions promoting self-associations.^21,22^

Caffeine exhibits a solubility in water of 0.1 mol kg^−1^, making it poorly water soluble due to self-stacking, despite being a relatively polar molecule exhibiting an octanol/water partition coefficient of log(Pow) = −0.07.^21,23,24^

To optimize caffeine extraction, one well known organic solvent is dichloromethane, particularly used in order to produce decaffeinated coffee.^25^ The use of conventional and classically used organic solvents is hampered by the increasing quest for solutions in line with the idea of sustainable development and also by the need to integrate these extracted molecules into food-grade formulations.

In this purpose, numerous publications present new strategies to try to improve methylxanthine solubilities. Some focus on the use of cyclodextrin to prepare inclusion complex with methylxanthines.^26–28^ This solubilization strategy prevent caffeine self-association by including them inside a hydrophobic cavity. This increases the indirect solubility of the caffeine, meaning that the numbers of monomers will be higher than in an aqueous solution of pure caffeine. Other techniques have been tried like the use of salts without showing a high efficiency.^29^ Supercritical CO_2_ and deep eutectic solvents have also been explored to extract caffeine.^30,31^ Finally, recent publications showed that the use of hydrotropic compounds can lead to efficient solubility of caffeine.^23^

Hydrotropes are water-soluble molecules that interact with poorly water-soluble compounds, significantly increasing their solubility in water. Several natural compounds, such as polyphenols exhibit hydrotropic properties.^32^ Therefore, solubility increases of such methylxanthines in water using hydrotropes are relevant for both extraction and formulation purposes, as the higher the solubility, the better the extraction and the more concentrated a homogenous solution is. Therefore, we aim at proposing here new sustainable extraction processes for methylxanthine compounds meant to be applied for food, nutraceuticals, cosmetics or environmental applications like insecticides by developing efficient solvents compatible with these applications. To investigate the application of methylxanthine compounds in aqueous formulation, we focused on aqueous solutions of natural polyphenol salts used as solubilizers. Such compounds are molecules known to present interesting biological properties and to be often used in many of the applications described above. To date, no systematic study has been carried out on such systems and, moreover, the specific molecular interactions between methylxanthine and polyphenols remain to be finely elucidated to explain their solubilization in aqueous solutions. To that end, we will present results on the measurement of solubilities of methylxanthine compounds in aqueous solutions at different concentrations of selected solubilizing agents, namely salicylate, ferulate, caffeate, nicotinate salts and niacinamide.

**Fig. 1:**
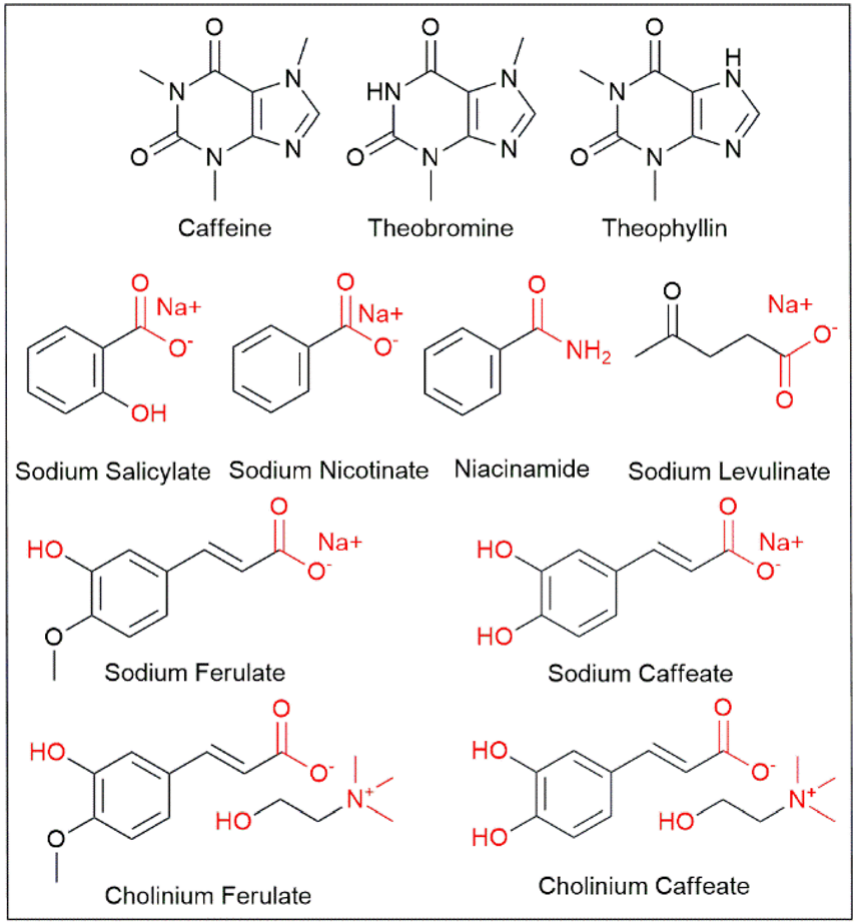
Chemical structures of compounds used in this study. Hydrophilic functionalities are represented in red.

Sodium salicylate (Fig. 1) is a model hydrotrope molecule.^33^ It occurs in many types of fruits, vegetables and spices and it has been shown that sodium salicylate can improve the growth of coffee plants.^34^ Vraneš *et al.* have already used it to enhance the solubility of caffeine.^23^ Sodium salicylate is a widely used compound with diverse applications across multiples industrial fields. It is often and particularly used in pharmacology to enhance the solubility of poorly water-soluble compounds. In food, it serves as a preservative and flavouring agent.^35^ Additionally, it finds extensive use in the cosmetic industry, as a pH regulator, anti-inflammatory agent, and exfoliant in skincare formulations.^36–38^ However, concerns arise regarding its potential to induce adverse reactions. Prolonged or excessive consumption of foods containing sodium salicylate may contribute to gastrointestinal discomfort and exacerbate existing gastrointestinal conditions.^39^ Applied on skin, it can lead to skin irritation and sensitization.^40,41^

Niacinamide and sodium nicotinate (Fig. 1) are natural products, also known as B3 vitamins and found in coffee plants.^42,43^ Their use as hydrotrope for caffeine solubilization in water has been reported by Park^44^*et al.* for niacinamide and by Román-Montalvo *et al.* for nicotinic acid using its cholinium salt.^45^ They are essential for maintaining healthy skin, nerves, and digestive system function.^46–48^ Niacin is also known to reduce the risk of cardiovascular disease.^49^ Moreover, niacin's antioxidant properties may confer protective effects against oxidative stress and inflammation.^50,51^ High doses of niacin, however, particularly in the form of supplements, can lead to adverse effects such as flushing, itching, and skin rash.^52^ Niacin high dose supplementation may also result in gastrointestinal symptoms such as nausea, vomiting, diarrhea and hepatotoxicity.^53^

Ferulic acid and caffeic acid (Fig. 1) are two molecules also present in coffee at reasonable amount and in a lot of other plants and fruits.^54–57^ They are known to have multiples health benefits and are already widely used in cosmetic industry for their antioxidant anti-aging or anti-bacterial properties.^28,58,59^ Considering their low solubility in water and according to some results recently published, these compounds have been used as sodium or choline salts. This gives the advantage to enhance their water solubility and conserving the antioxidant properties of these compounds, as it is conferred by the substituents on the aromatic ring of these molecules.^60^ The preparation of cholinium salts of ferulate and caffeate has already been report in literature and characterized previously. In addition to increasing the solubility of phenolic acids, it also enhances their antioxidant properties.^32,61,62^

In the present paper, the modification of water solubility of caffeine, theobromine and theophylline by addition of different polyphenol salts was studied hoping that they act as hydrotropes for the three methylxanthines considered. The solubilizing efficiency of sodium and cholinium salts of caffeate and ferulate, sodium nicotinate and sodium salicylate, as well as the well-known hydrotrope niacinamide has been tested. Because all compounds studied here exhibit an aromatic ring, a non-aromatic compound, namely sodium levulinate, has also been used for comparison reasons. To the best of our knowledge, there has been no report on the use of sodium and cholinium ferulate and caffeate for improving the solubility of methylxanthines in water while these compounds are naturally occurring in coffee and have advantageous activities on health. Similarly, 2D NMR and molecular dynamics of such aqueous solutions have not been reported previously. Beyond improving solubility values, understanding the molecular origin of methylxanthine solubilization is critical to design sustainable and efficient solubilizing solutions. Here, we combine systematic solubility measurements with ROESY NMR and molecular dynamics simulations to establish a clear structure-solubilization relationship for a series of naturally derived aromatic salts, enabling a mechanistically supported and providing guidelines for the selection of natural and efficient aqueous solubilizers of methylxanthines.

## Materials and methods

### Chemicals

Caffeic acid (CAS 331-39-5) >98% TCI, ferulic acid (CAS 537-98-4) >98% TCI, sodium salicylate (CAS 54-21-7) >99,5% have been purchased from SIGMA, sodium nicotinate CAS 54-86-4 >98% from TCI, niacinamide CAS 98-92-0 >98% from Alfa Aesar, choline hydroxide 46 wt% in water, CAS 123-41-1 from Sigma-Aldrich, sodium hydroxide 1 M ± 0.2% in water from ROTH, caffeine CAS 58-08-2 >98% from TCI, theobromine 83-67-0 >98% from TCI, theophylline CAS 58-55-9 >98% from TCI , levulinic acid CAS 123-76-2 98% from Sigma-Aldrich. Chemicals were used as received.

## Methods

### Sample preparation of sodium salicylate, niacinamide and sodium nicotinate

For each hydrotropic agent, aqueous solutions were prepared over a concentration range spanning from 0.1 to 3 mol kg^−1^. The appropriate mass of solute was accurately weighed with a Entris II Sartorius precision scale and added to 5 g of MilliQ water in sealed glass vials. The mixtures were stirred magnetically at room temperature (22 °C, 500 rpm) until complete dissolution, typically within 10–20 minutes. All samples were protected from light throughout the preparation and used within the same day.

The actual molalities of the hydrotropes were determined *a posteriori* by quantitative ^1^H NMR spectroscopy, using the water signal as an internal reference. This approach ensured accurate concentration determination, independent of minor deviations arising during sample preparation.

### Sample preparation of sodium polyphenolate in water

Aqueous solutions of sodium caffeate and sodium ferulate were prepared over a concentration range of 0.05 to 0.40 mol kg^−1^ according to previously reported procedure by neutralizing the corresponding phenolic acids with a hydroxide salt.^32^ All chemicals were commercially available and used as received without further purification. Phenolic acids were first weighed using a precision analytical scale and introduced into glass vials and added to 5 g of MilliQ water to which a stoichiometric amount of a freshly prepared 1.0 M sodium hydroxide solution was first added.

The resulting mixtures were magnetically stirred at room temperature in sealed vials protected from light until complete dissolution and full neutralization of the acids into their corresponding sodium salts. The pH of the final solutions was measured to confirm complete salt formation. Solutions were used right after neutralisation, without removing water nor carrying out any further purification. The actual molalities of the solubilizing agents were determined *a posteriori* by quantitative ^1^H NMR spectroscopy, using the water signal as an internal reference, ensuring accurate concentration values irrespective of minor preparation deviations.

### Sample preparation of cholinium polyphenolate in water

Phenolic acids were weighed using a precision analytical scale and introduced into glass vials. All chemicals were commercially available and used as received. An aqueous solution containing an equimolar amount of choline hydroxide (46 wt% choline hydroxide in water) was first added, followed by additional deionized water to reach a total water mass of 5 g. Neutralization and dissolution were carried out under magnetic stirring at room temperature in sealed vials protected from light until complete salt formation. The pH of the resulting solutions was measured to confirm full neutralization. Cholinium salts aqueous solutions were used directly after preparation without any further purification.^32,62^ The actual molalities of the solubilizing agents were determined *a posteriori* by quantitative ^1^H NMR spectroscopy, using the water signal as an internal reference, ensuring accurate concentration values irrespective of minor preparation deviations.

### Solubility of caffeine, theophylline and theobromine

All solubility experiments were carried out at room temperature (22 ± 2 °C) In a glass vial containing typically 2 g of an aqueous solution of the solubilizing agent prepared as described above, approximately 30 mg of the methylxanthine were added stepwise. After each addition, the vials were placed on a stirring plate and stirred for approximately 15 min. If complete dissolution of the methylxanthine was observed, as evidenced by a clear solution, an additional 30 mg portion was added, and the mixture was stirred again for 15 min. When the formation of a precipitate was observed, the vial was maintained under stirring conditions for an extended equilibration period of 12 h at 450 rpm to ensure equilibrium was reached. Solubility was considered attained when undissolved solid remained visible after this 12 h equilibration step. After equilibration, the samples were centrifuged for 10 min at 14 000 rpm. The supernatant was then collected and filtered through 0.22 µm PTFE syringe filters prior to quantitative analysis by ^1^H NMR spectroscopy. Each solubility experiment was performed in triplicate. Reported solubility values correspond to the average of three independent measurements, and error bars represent the associated standard deviation.

### 1H NMR spectroscopy

Samples (0.8 mL) were transferred into NMR tubes prior to analysis. All NMR measurements were performed in the dark at 298 K. Proton nuclear magnetic resonance (^1^H NMR) spectra were acquired on a Bruker Avance III HD 400 spectrometer operating at a proton frequency of 400.13 MHz.

Spectra were recorded in H_2_O using a relaxation delay of 2 s and 64 scans. Data processing was carried out using MestReNova software, with automatic phase and baseline corrections applied prior to analysis. Water signal was set to 4.80 ppm. The isolated one-proton signal of caffeine was first integrated and set to 1, providing the normalization factor for all other integration of signals. For each solubilizing agent, the average value of several well-resolved one-proton signals was calculated. The water signal was divided by 2 to account for its two protons.1
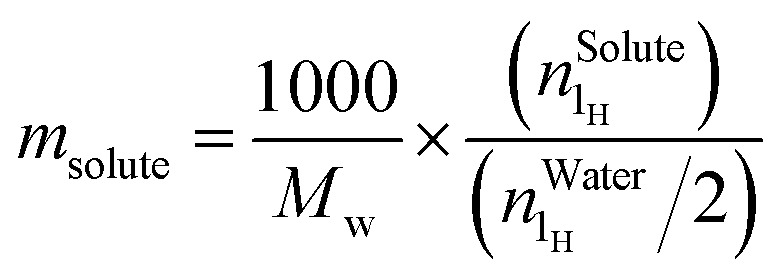
with *m*_solute_ being the molality of either methylxanthine or a solubilizing agent, expressed in mol kg^−1^ of water, *M*_W_ the molecular weight of water, 
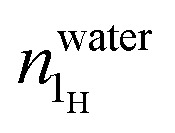
 the number of protons as obtained from integration of the peak at 4.80 ppm and 
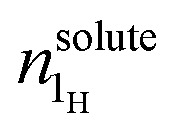
 the number of protons of methylxanthine or a solubilizing agent, as obtained from integrating peaks corresponding to each compound. In the case of methylxanthine, 
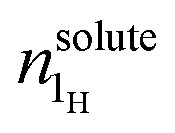
 is set to 1, as explained above. Each NMR measurement was performed on independently prepared samples.

### 2D ROESY spectroscopy

Nuclear Magnetic Resonance (NMR) studies were conducted using a Bruker Avance III HD 400 MHz spectrometer. Given the challenge of estimating the optimal mixing time to determine the most populated structure of intermolecular complexes using the Nuclear Overhauser Effect (NOE), Rotating Frame Overhauser Effect Spectroscopy (ROESY) was employed. Due to the low concentration of the components under study, the experiments were optimized for sensitivity rather than precise intermolecular distance measurements.^63^ The optimized conditions included 0.6 mL D_2_O samples, a mixing time of 0.8 s, a relaxation delay of 3 s, and 64 scans. Spectra were processed with MestReNova. Among caffeine–caffeine interactions, the [A–B] cross-peak, between the methyl group A and the adjacent C–H proton B, shows the highest intensity, unsurprisingly due to both strong spatial proximity and intra-molecular scalar *J*-coupling. Therefore, this cross-peak was chosen as the normalization reference (intensity = 1.0) across all samples.

Spectra were acquired once for each most concentrated solutions obtained for each aromatic hydrotropes and only with caffein due to the weak concentration of the other methylxanthines studied here.

### Density measurements

Density measurements were conducted on a density meter DMA 4500 from Anton Paar. Measurements were carried out at 25 °C. Blank samples were measured prior to measurements with air and pure water. Density of samples were measured adding typical 0.8 mL into the vibrating tube of the densimeter. Densities are given ± 0.0001 g.cm^−3^. All samples were measured in triplicates.

### Computational study

Molecular dynamics simulations were performed using Amber18 with the Gaff2 force field for organic molecules.^64^ Initial modelling boxes were prepared using the Packmol Package to obtain cubic boxes with sides of 80 Å.^65^ Charge parameters of the organic compounds were parametrized using Gaussian16 following the RESP procedure.^66,67^ All used atoms in the simulation box were treated explicitly. Periodic boundary conditions were applied, and the chosen integration step size was of 2 fs. Each molecular dynamics simulation underwent several preparation phases before the production phase: energy minimization was carried out to prevent high-energy structures and steric clashes; heating phase lasted for 50 ps; NVT equilibration was ran for 100 ps and NPT equilibration for 500 ps. The systems were then sampled for 300 ns long trajectories. Calculated densities of our systems correlate perfectly with experimentally measured ones which leads us to be confident in the validity of our models.

## Results

### Solubility of methylxanthine in water

**Fig. 2:**
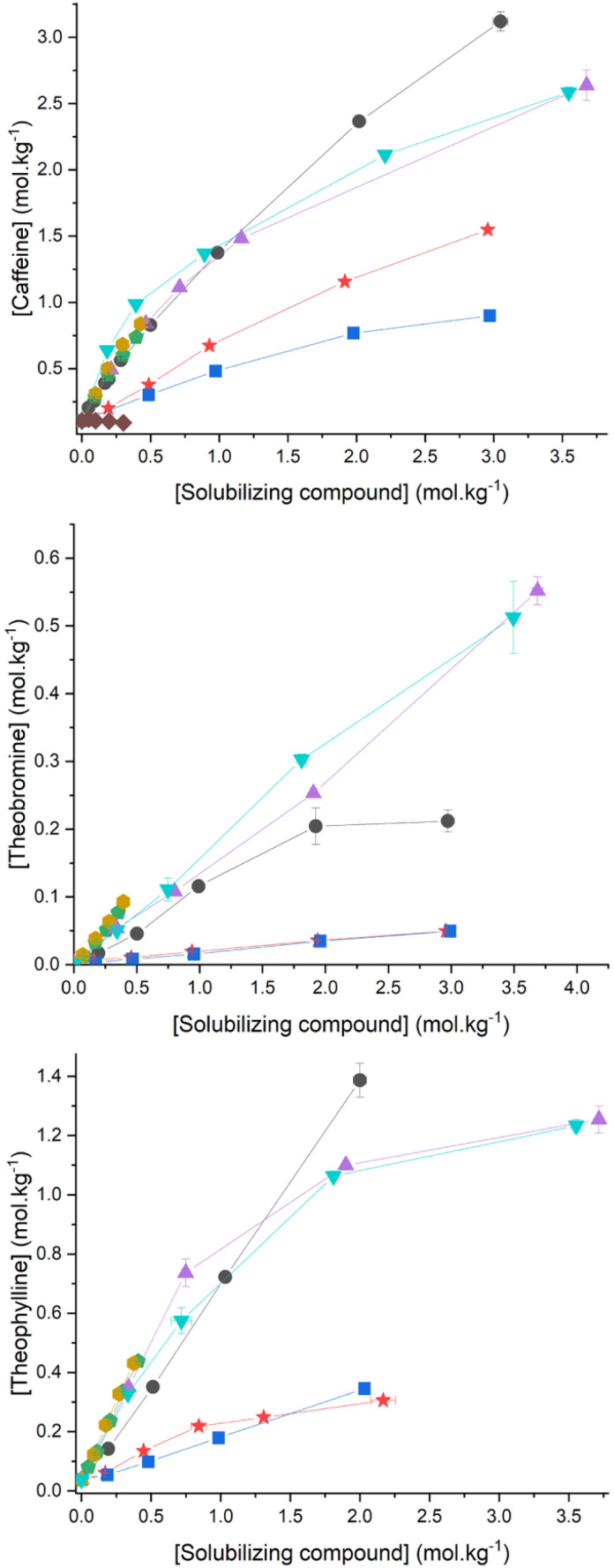
Methylxanthine solubility in presence of solubilizing compounds. Left: solubility of caffeine in water. Center: solubility of theophylline in water. Right: solubility of theobromine in water. ●: sodium salicylate, ▼: cholinium caffeate. ▲: cholinium ferulate. □: sodium caffeate. □: sodium ferulate. ★: niacinamide. ■: sodium nicotinate. ◆: sodium levulinate.

As detailed in the experimental section, solubilities in water for caffeine, theophylline and theobromine were measured as a function of concentration of various solubilizing agents, namely sodium salicylate, sodium and cholinium ferulate, sodium and cholinium caffeate, niacinamide and sodium nicotinate. Solubilities of all three methylxanthines are plotted as a function of the concentration of each solubilizing agent in Fig. 2.

Our study was limited to solutions containing up to 3.0 mol kg^−1^ of sodium salicylate because of a gel formation obtained at higher concentrations, which is a known phenomenon of salicylate salts in solution, as reported by Vinkx *et al.* with starch.^68^ Even though no such gel formation was observed when using cholinium ferulate or caffeate, accordingly, we limited our study to solutions containing up to 3.5 mol kg^−1^ to avoid increase in viscosity and experimental difficulties in handling such solutions. It is worth noticing that the solubility in water of sodium caffeate and sodium ferulate is, respectively, 0.37 and 0.41 mol kg^−1^, which was an initial concentration limit of our study, as shown in Fig. 2.^32^ Such significant increase in the solubility in water of cholinium alkylate salts compared to sodium salts have already been reported previously.^69^

In pure water, solubilities for caffeine, theophylline and theobromine were found to be 0.106 mol kg^−1^, 0.038 mol kg^−1^ and 0.002 mol kg^−1^, respectively. It first appears that solubilities in water of these three methylxanthines, increased significantly in presence of all tested solubilizing agents. Values as high as 3.1 mol kg^−1^ of caffeine and 1.4 mol kg^−1^ of theophylline were obtained in an aqueous solution of 3.0 mol kg^−1^ and 2.0 mol kg^−1^ of sodium salicylate, respectively. A value of 0.55 mol kg^−1^ for theobromine water solubility was obtained in presence of 3.6 or 3.7 mol kg^−1^ sodium caffeate or sodium ferulate, accordingly. In the case of caffeine, solubility in water increases linearly and almost similarly in presence of up to 0.4 mol kg^−1^ of ferulic or caffeic acid and up to 1 mol kg^−1^ of either sodium salicylate, ferulate and caffeate. For the same anion, there seems to be a negligible influence of H^+^ or Na^+^ on the solubility of caffeine.

Above 1 mol kg^−1^ of solubilizing agent, the solubility curve of caffeine in presence of sodium salicylate is higher than any other curve and is nearly linear over the whole concentration range. In presence of sodium caffeate or ferulate, solubility curves exhibit an inflection point at 1 mol kg^−1^, reaching a value of approximately 2.55 mol kg^−1^ for the water solubility of caffeine in presence of 3.5 mol kg^−1^ choline caffeate or choline ferulate. The influence of niacinamide and sodium nicotinate on the solubility in water of caffeine is lower than that of salicylate, ferulate and caffeate salts. Solubility curves are almost linear and reach values of 1.5 mol kg^−1^ and 0.75 mol kg^−1^ caffeine in 3.0 mol kg^−1^ niacinamide and sodium nicotinate, respectively. Finally, sodium levulinate has no influence or even a negative effect on the aqueous solubility of caffeine.

In the case of theophylline, solubility curves in presence of sodium salicylate are also linear, exhibiting a maximum value of 1.4 mol kg^−1^ at 2.0 mol kg^−1^ sodium salicylate. Surprisingly, salts based on caffeate and ferulate appear to be better at solubilizing theophylline in water than sodium salicylate, at least below 0.7 mol kg^−1^ of solubilizing salt. At higher concentration, an inflection in solubility curve is observed. Once again, niacinamide and sodium nicotinate are the least efficient at solubilizing theophylline.

Finally, in the case of theobromine, most efficient solubilizing agents are caffeate and ferulate salts. Up to 0.4 mol kg^−1^, ferulic acid and caffeic acid are more efficient than their sodium homologues. Cholinium caffeate and choline ferulate have a similar influence on the solubility of theobromine. Solubility curves are linear and reach a maximal value of 0.55 mol kg^−1^ theobromine in 3.7 mol kg^−1^ cholinium ferulate. Surprisingly, in presence of sodium salicylate, a plateau in the solubility of theobromine is observed at 2 mol kg^−1^ of solubilizing agent.

To investigate further the influence of solubilizing agents on the solubility of a methylxanthine, solubilities of methylxanthines measured here were compared to their value in pure water. To that end, a solubility increase factor, denoted as *F*_S.I._, was calculated as follows:2
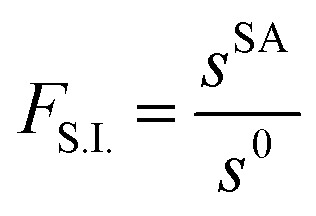


With *s*^SA^ and *s*^0^ the solubilities of methylxanthine in presence of a solubilizing agent and in pure water, respectively. As stated above, solubilities of caffeine, theophylline and theobromine are 0.106, 0.038 and 0.0018 mol kg^−1^ of water, respectively.

**Fig. 3:**
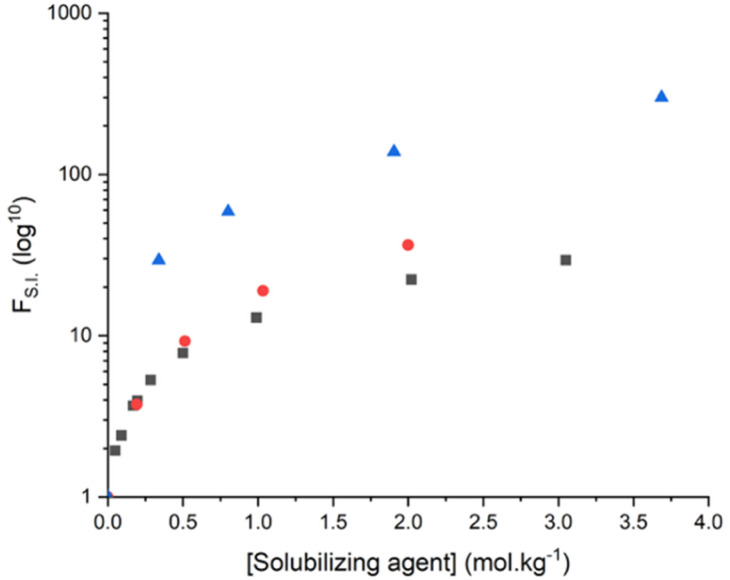
Solubility increase factor, *F*_S.I_, for caffeine, theophylline and theobromine added into aqueous solutions containing sodium salicylate or cholinium ferulate. ▲: theobromine and cholinium ferulate, ●: theophylline and sodium salicylate, ■: caffeine and sodium salicylate.

For each methylxanthine studied here, the solubility increase factor has been plotted in Fig. 3 as a function of the molality of the solubilizing agent yielding highest *F*_S.I_ values.

This corresponds to plotting *F*_S.I_ values for caffeine and theophylline as a function of sodium salicylate concentration and for theobromine as a function of cholinium ferulate concentration, respectively.

Results show that for the same solubilizing agent, the solubility increase of theobromine is always higher than those of caffeine or theophylline, accordingly. A maximum *F*_S.I_ value of 301 is obtained for theobromine in aqueous solutions of 3.7 mol kg^−1^ of cholinium ferulate. Caffeine appears to be the methylxanthine molecule exhibiting the lowest solubility increase in presence of sodium salicylate. This is explained by the solubility in water of caffeine that is non negligible, hence a solubility increase being limited.

To facilitate comparison between all systems the best conditions to solubilize each studied methylxanthines in this study were summarize in Table 1.

**Table 1:** Best conditions to solubilize each methylxanthines

Methylxanthine	Solubilizing agent	[Solubilizing agent] mol kg^−1^	Solubility mol kg^−1^
Caffeine	Sodium salicylate	3	3
Theophyllin	Sodium salicylate	2	1.4
Theobromine	Cholinium ferulate	3.7	0.55

### Water-caffeine-sodium salt phase diagram

Because caffeine has already been reported to exhibit hydrotropic properties, hence having a potential influence on the solubility of ferulic and caffeic acid in water, a set of experiments were conducted at high concentrations of sodium ferulate or caffeate, accordingly, above the reported solubility of 0.4 mol kg^−1^ previously reported for these two salts in water.^32^

**Fig. 4:**
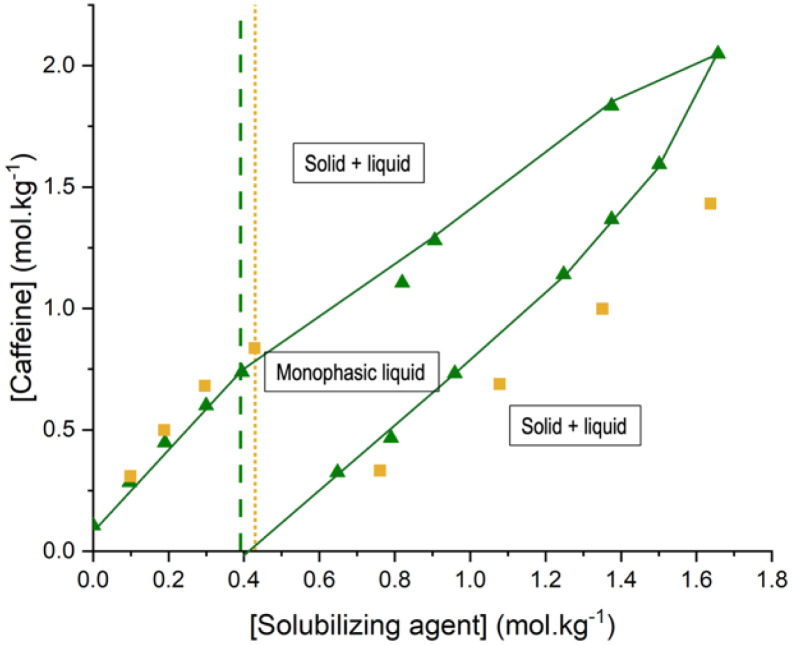
Phase diagram for aqueous solutions containing caffeine and sodium ferulate or sodium caffeate, accordingly: ■ solutions containing sodium caffeate. ▲: solutions containing sodium ferulate. Green dashed line: solubility limit of sodium ferulate in pure water. Yellow dashed line: solubility limit of sodium caffeate in pure water green full line: guide for the eyes.

To that end, several experiments were carried out adding caffeine into a water saturated mixture of sodium ferulate or caffeate and a given amount of the corresponding precipitated sodium salt. Strikingly, upon addition of caffeine, the amount of sodium ferulate precipitate was found to gradually decrease until complete dissolution, yielding a homogeneous solution, exhibiting a slightly yellow color and low viscosity. Further addition of caffeine eventually led to the formation of a new precipitate, most probably corresponding to pure caffeine. Repeating this experiment with various amounts of sodium caffeate or sodium ferulate led to a phase diagram, presented in Fig. 4.

Three domains are clearly identified in Fig. 4. Curves are guides to the eye and correspond to so-called binodal curves, delimitating two domains on a phase diagram.

First domain observed in Fig. 4 is the one located in the center of the diagram, limited by the two binodal curves. This domain describes all homogeneous solutions of caffeine and either sodium caffeate or sodium ferulate. The upper binodal curve of this domain represents solutions of sodium ferulate or caffeate saturated in caffeine. Above this curve, caffeine precipitates from the solution. The lower binodal curve corresponds to homogeneous solutions of caffeine saturated in sodium caffeate or ferulate, respectively.

Strikingly, the phase diagram shows that homogeneous solutions can be obtained at concentration of sodium caffeate or ferulate well beyond their solubility limit in pure water. For example, under our experimental conditions, a clear aqueous solution containing a 1.65 mol kg^−1^ of sodium ferulate was obtained in presence 2 mol kg^−1^ of caffeine, a value significantly higher than that of 0.41 mol kg^−1^ for sodium ferulate in pure water. These experiments reveal a significant mutual solubilizing effect between a solubilizing agent and caffeine, extending significantly the domain of solubility of a given metabolite and of a specific solubilizing agent.

These results open the way to two distinct strategies for dissolving a metabolite in an aqueous solution of polyphenol. On the one hand, one can form a choline salt thereof, the latter exhibiting a very high solubility in water. On the other hand, one can simply form the sodium or potassium salt thereof. In the latter case, although the solution can easily reach saturation, addition of a second aromatic molecule can overpass this limitation and significantly enhance the solubility.

### NMR studies

**Fig. 5:**
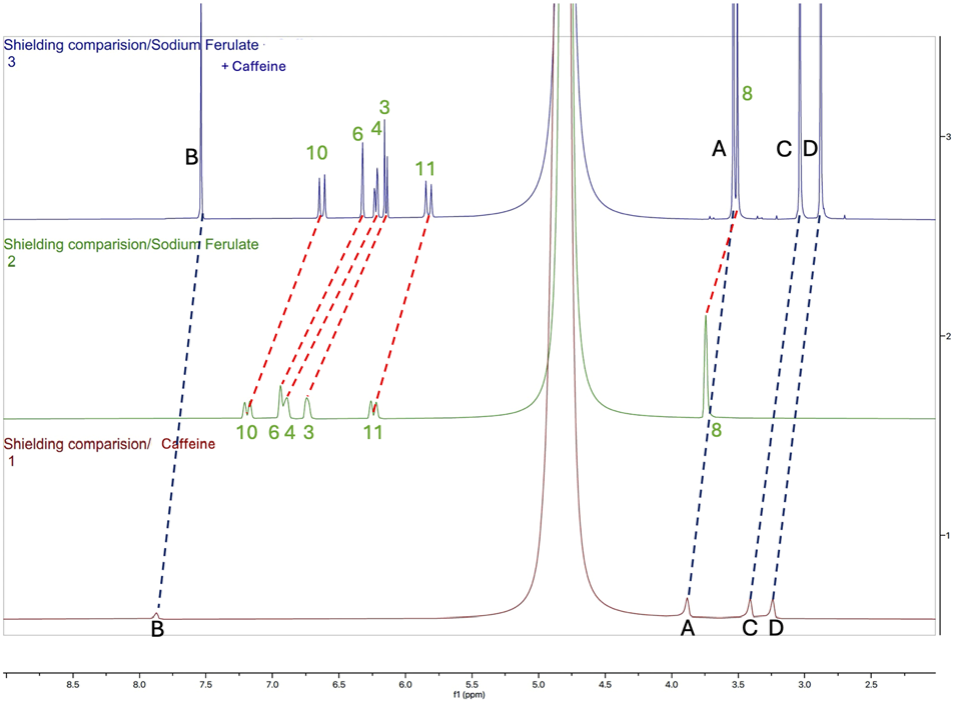
Comparison of ^1^H NMR shielding of sodium ferulate, caffeine, and their mixture in water. Caffeine (red spectrum), sodium ferulate (green spectrum), caffeine/sodium ferulate mixture (blue spectrum).

To gain insights into the intermolecular interactions occurring in such aqueous solutions, ^1^H NMR spectra were recorded for aqueous solutions of caffeine, sodium ferulate, and corresponding mixtures thereof. 1H NMR spectra are shown in Fig. 5. A noticeable shielding of all protons of sodium ferulate and caffeine in the mixed sample is observed, compared to those observed in aqueous solutions containing either caffeine or sodium ferulate. Moreover, such a shielding effect is more pronounced for the protons of the aromatic ring of ferulate (Δ_ppm_ ∼0.6) than those on the three carbons outside the aromatic ring going from the nearest proton from the aromatic ring with a Δ_ppm_ of ∼0.56 to the farthest one with a Δ_ppm_ of ∼0.41. The lowest shift stands for the proton located on the methoxy group around the aromatic cycle with a value of Δ_ppm_ of 0.24. Such shielding has been previously reported and linked to the formation of a complex between molecules.^70,71^

**Fig. 6:**
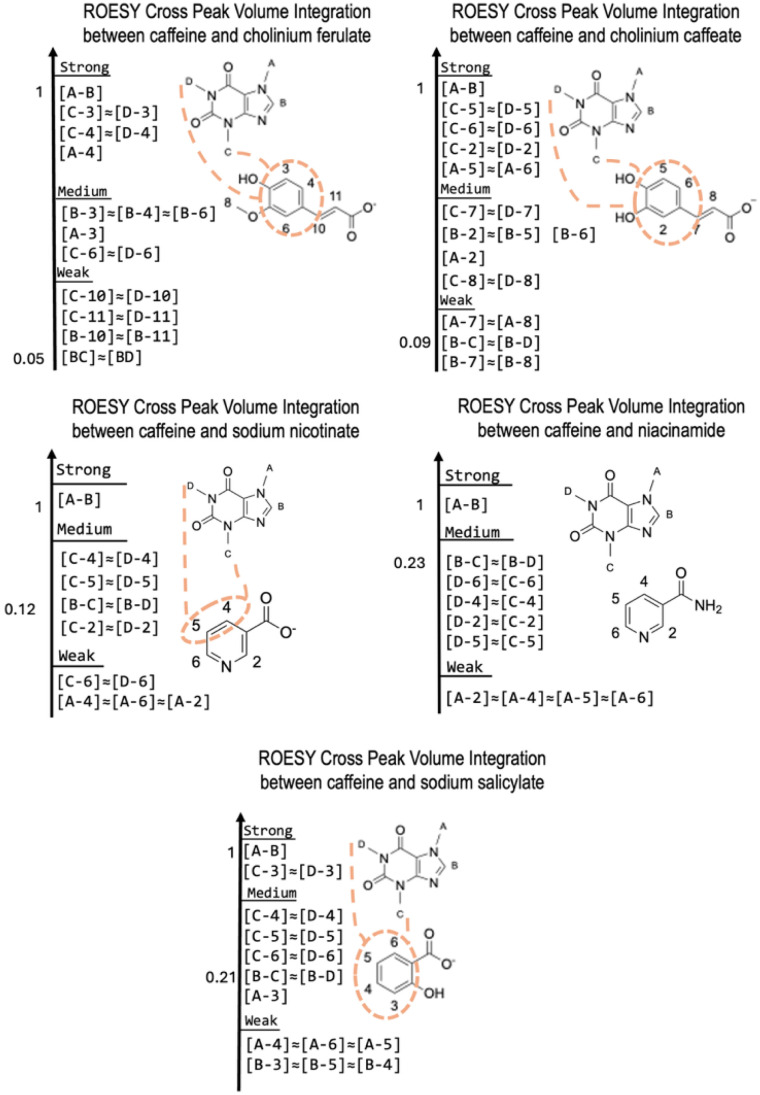
ROESY cross-peaks volume integration ranking for 3 mol kg^−1^solutions of solubilizing agent saturated with caffeine. Highest cross-peaks volume integration corresponding to most probable occurring spatial closing in each sample is marked by orange lines.

To gain deeper insights into the location and intensity of intermolecular interactions between caffeine and the different solubilizing agents, 2D-NMR ROESY spectroscopy was employed on selected aqueous solutions. These included the most concentrated caffeine solutions achievable for each system, with caffeine concentrations ranging from approximately 1 to 2.5 mol kg^−1^. The complexing agents (CA) studied were cholinium caffeate, cholinium ferulate, sodium salicylate, sodium nicotinate, and niacinamide, each present at concentrations around 3.0 to 3.4 mol kg^−1^. All ROESY spectra are collected in Fig. S.7–S.11 of the supplementary information file. Results are presented in Fig. 6 as normalized cross-peak volume integrations. These intensities reflect the time-averaged spatial proximity between specific proton pairs, rather than precise interatomic distances, due to the long mixing times used in 2D-NMR ROESY acquisition.

2D-NMR ROESY cross-peaks are denoted as [*X*–*Y*], where *X* and *Y* represent specific proton sites. Each proton from caffeine is assigned a letter (A, B, C and D), while those on complexing agents are designated with numbers (2–11). Full proton assignments are provided in Fig. 6. Cross-peaks between two caffeine's protons are described as [A–B], [B–C], and [B–D]. Among caffeine–caffeine interactions, the [A–B] cross-peak, between the methyl group A and the adjacent C–H proton B, shows the highest intensity, unsurprisingly due to both strong spatial proximity and intra-molecular scalar *J*-coupling. Therefore, this cross-peak was chosen as the normalization reference (intensity = 1.0) across all samples. All other cross-peaks were then compared relative to this reference, providing a consistent scale for evaluating the strength of through-space dipolar interactions. Intermolecular spatial proximities between caffeine molecules are reflected in the [B–C] and [B–D] cross-peaks as intramolecular spatial interactions and *J*-coupling between these methyl groups are expected to be negligible. These are thus purely indicative of spatial proximity between separate molecules. Moreover, it is important to consider that the likelihood of forming such pairs correlates with the concentration of caffeine in the solution.

In the samples containing cholinium caffeate and cholinium ferulate, where caffeine concentration reached approximately 2.5 mol kg^−1^, [B–C] and [B–D] cross-peaks were the weakest among all systems studied, with normalized volume integration of 0.09 and 0.05, respectively. This suggests that, despite the high caffeine concentration, homo-association (caffeine-caffeine) is strongly disfavoured in favour of hetero-association with the solubilizing agent. Furthermore, it appears that cross-peaks between caffeine protons and protons located on the aromatic ring on caffeate or ferulate anions exhibit stronger intensities than those with protons located out of the aromatic ring. This suggests that the aromatic ring of caffeate and ferulate is more involved in caffeine-solubilizing agent interaction, and hence most probably plays a significant role in solubilizing caffeine in water.

Similar results are obtained with sodium salicylate. In this case both caffeine and sodium salicylate concentrations were around 3 mol kg^−1^ and the normalized volume integration for the [B–C] and [B–D] cross-peaks is relatively high (0.21). Nonetheless, all cross-peaks involving the aromatic ring protons of salicylate and protons on the pyrimidine ring of caffeine exhibit even greater intensity. This suggests that, like the mixtures with cholinium ferulate and cholinium caffeate, caffeine molecules predominantly form hetero-associations with complexing agent molecules rather than homo-associations between caffeine molecules.

The normalized volume integrations of the [B–C] and [B–D] cross-peaks in the caffeine-sodium nicotinate sample are 0.12. However, in this mixture, the caffeine concentration is below 1 mol kg^−1^, which is three times lower than the concentration of the complexing agent. Despite this, only four caffeine-sodium nicotinate cross-peaks, namely [C-4], [C-5], [D-4] and [D-5], are above this value. This observation strongly suggests that, in this mixture, caffeine molecules preferentially form homo-associations rather than hetero-associations.

Finally, the highest normalized volume integration values for the [B–C] and [B–D] cross-peaks, reaching 0.23, were observed in the caffeine/niacinamide sample. These values surpass the intensities seen for all caffeine-niacinamide cross-peaks. In this mixture, the caffeine concentration is approximately 1.5 mol kg^−1^, which is half the concentration of the complexing agent. Evidently, like the mixture with sodium nicotinate, caffeine molecules preferentially form homo-associations rather than hetero-associations.

This confirms that in presence of all solubilizing agents studied here, caffeine interacts significantly with caffeate or ferulate, accordingly, and to a lower extent to salicylate, rather than sodium nicotinate and niacinamide.

This also reveals that protons on a benzene ring interacts with protons on caffeine in a stronger way than those located on pyridinium ring. These results correlate well, at least qualitatively, with the solubilities in water of caffeine measured in presence of solubilizing agents and presented in Fig. 2. One can there infer that interactions between caffeine and say, caffeate or ferulate anions, are related to a proximity between caffeine and the aromatic ring of these anions.

### Molecular dynamics

**Fig. 7:**
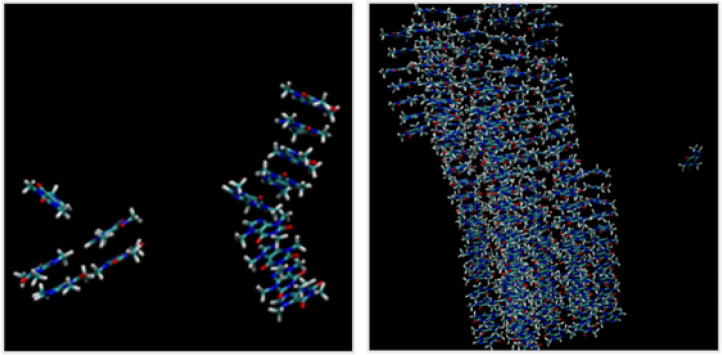
Snapshots of the last frame of 300 ns molecular dynamics simulation of aqueous solutions of caffein. Left: 0.1 mol kg^−1^ caffeine. Right: 0.8 mol kg^−1^ caffeine aqueous solution. Water molecules are omitted.

In order to obtain information on the relative positioning of the solubilizing agents, as well as their number, around a single caffeine molecule, numerous molecular dynamics simulations were carried out. These “all atom” simulations will complement the insights brought by NRM experiments by depicting the way solubilizing agents interacts with caffeine giving rise to a general pattern of caffeine solubilization mechanism. To that end, two initial simulations were performed to describe aqueous caffeine solutions: the first, at a concentration of 0.106 mol kg^−1^, simulates the behavior of caffeine in water at its solubility limit, and the second, at a concentration of 0.83 mol kg^−1^, corresponds to an oversaturated aqueous solution. Snapshots are shown in Fig. 7.

At 0.1 mol kg^−1^ of caffeine, that is slightly below its solubility in water, caffeine appears to form small aggregates in water, but no precipitate. On the opposite, at 0.83 mol kg^−1^ of caffeine in water and starting from a randomized molecular spatial distribution, caffeine molecules rapidly aggregate into fibrils *via* stacking interactions, as shown in Fig. 7.

Subsequently, simulation boxes containing water, caffeine, and 0.1 mol kg^−1^ solubilizing agent were prepared. Values of the concentration of caffeine in such solutions were set to values slightly below those of the solubility curves presented in Fig. 2, a concentration, at which a monophasic clear solution is observed experimentally. Please note that, as shown in Fig. 1, at a concentration of 0.1 mol kg^−1^, sodium caffeate and sodium ferulate were found to dissolve an amount of caffeine slightly higher than that dissolved using 0.1 mol kg^−1^ sodium salicylate.

**Fig. 8:**
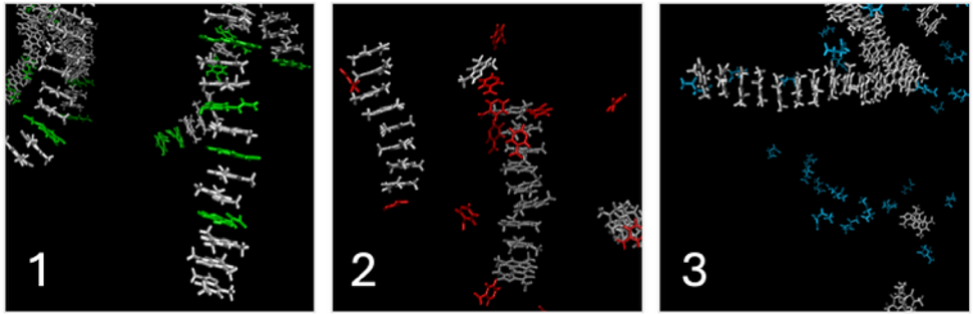
Snapshots of the last frame of a 300 ns molecular dynamics simulation of an aqueous solutions of caffeine in presence of selected solubilizing agents. Caffeine is always presented in white. Snapshot 1 : 0.25 mol kg^−1^ caffein and 0.1 mol kg^−1^ sodium caffeate (drawn in green). Snapshot 2 : 0.15 mol kg^−1^ caffeine and 0.1 mol kg^−1^ sodium nicotinate (drawn in red). Snapshot 3 : 0.1 mol kg^−1^ caffeine (white) and 0.1 mol kg^−1^ sodium levulinate (drawn in blue).

Selected snapshots for solutions of caffeine containing sodium caffeate, sodium nicotinate and sodium levulinate are presented in Fig. 8. Snapshots of all other systems are available in SI.

Taking into account sodium caffeate, sodium nicotinate or sodium ferulate in the systems leads to significantly different results. In presence of caffeate anions, fibrils are observed as in the case of 0.1 mol kg^−1^ of caffeine in water. Such fibrils are, however, composed of caffeine and caffeate anions, the latter interacting between caffeine molecules. In the presence of sodium nicotinate, small aggregates of caffeine are observed, nicotinate anions being present at the top and bottom of aggregates. Finally, in the case of sodium levulinate, fibrils of caffeine like those shown in Fig. 8 are observed, levulinate anions not being in close contact with caffeine.

Aromatic compounds containing benzene rings interact more efficiently with caffeine, intercalating themselves within caffeine fibrils, whereas pyridine-based compounds primarily stacked at the extremities of these fibrils. This clearly tends towards a solubilization mechanism driven by π-stacking interactions. Sodium levulinate, due to the absence of aromatic ring, has no effect on the solubility of caffeine. Fig. 8 shows that molecular dynamics successfully described aqueous solution of neat caffeine while comparing our simulated results with experimental solubility and 2D NMR results.

The average numbers of caffeine and water molecules surrounding a single caffeine molecule throughout the simulations were quantified using a dedicated script. Results are presented in Table 2. As expected, at a concentration of caffeine above its solubility limit in water, a significant increase in caffeine molecules surrounding a central caffeine is observed, changing from 2 to 6 molecules, while the number of water molecules surrounding a central caffeine decreases from 13 to less than 8. In presence of complexing agents, even at concentrations above the solubility limit of caffeine in water, namely 0.106 mol kg^−1^, the number of caffeine molecules surrounding a central caffeine remained mostly constant. Similarly, the number of water molecules around caffeine remained stable, suggesting that aggregates formed between caffeine and solubilizing agent as observed in Fig. 8, remain soluble at such concentrations. As shown in Fig. 8, this is due to the integration of caffeate or ferulate into caffeine fibrils. Such a phenomenon appears to yield a fibril embedding several hydrophilic hydroxyl or ester groups on its surface, resulting in an enhanced dissolution thereof in water.

**Table 2:** Average number of caffeine and water molecules surrounding one caffeine molecule at different caffeine concentrations and with or without complexing agents

Caffeine (mol kg^−1^)	Solubilizing agent (0.1mol kg^−1^)	# Of caffeine surrounding one caffeine	# Of H_2_O surrounding one caffeine
0.106	—	2.12	13.38
0.83	—	6.22	7.7
0.25	Nacaffeate	1.61	13.0
0.25	Naferulate	1.36	13.25
0.22	Nasalicylate	2.1	12.95
0.15	Niacinamide	1.86	12.96
0.15	Nanicotinate	1.92	13.33

**Fig. 9:**
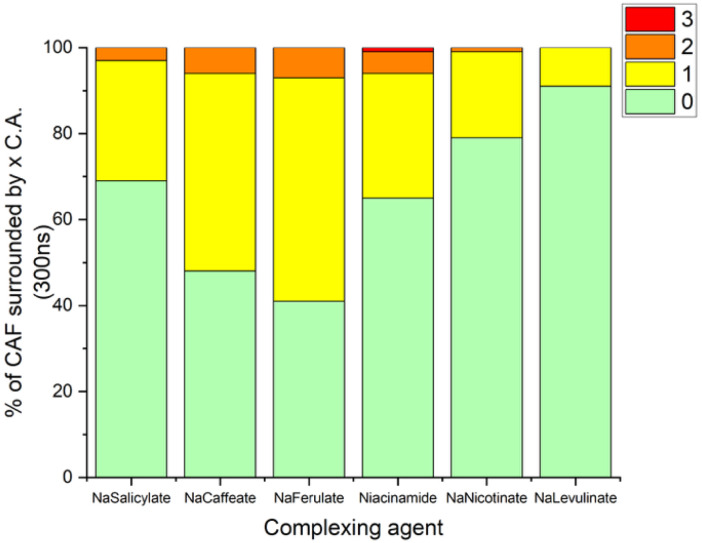
Percentage of caffeine molecule surrounded by a number of solubilizing agents (SA) calculated from a 300 ns M.D. Simulation for each SA. Green: no solubilizing agent is surrounding caffein. Yellow: (1) solubilizing agent is found around one caffein molecule. Orange: (2) solubilizing agents are found around one caffein molecule. Red: (3) solubilizing agents are found around one caffein molecule.

Further, for each solution, interactions between a complexing agent and caffeine fibrils were quantified by calculating the average amount of a given molecule, namely caffeine, solubilizing agent or water, respectively, around each central caffeine moiety. Results are reported in Fig. 9, showing the proportion of three different types of structures. Green bars represent caffeine surrounded by two caffeine neighbours above and below its equatorial plane. Yellow bars indicate a mix of caffeine and complexing agents above and below a caffeine molecule. Orange bars correspond to two complexing agents surrounding a caffeine molecule. Sodium ferulate and sodium caffeate appear the most efficient at integrating into caffeine fibrils, as evidenced by their higher proportions of mixed configurations, represented by yellow and orange segments in Fig. 9.

These findings are in good agreement with the solubility curves and 2D NMR data obtained in this study. Molecular dynamics simulations first confirmed that sodium levulinate has no significant effect on caffeine solubility, as no specific interactions were visually observed that could facilitate the solubilization of caffeine aggregates. Further, this compound appears to be the less keen on surrounding caffeine molecules, as shown on Fig. 9.

Second, heteroaromatic compounds such as niacinamide and sodium nicotinate were found to be less effective in disrupting caffeine fibrils than benzene-based aromatic molecules. Visual analysis of the simulations reveals that these molecules preferentially stack at the top and bottom of the fibrils rather than integrating into their structure, which agrees with our previous observations and results. However, this observation is not well captured in Fig. 9, as the diagram only shows the average number of complexing agents around each caffeine molecule, without considering how many caffeine molecules are present in each system. This introduces a bias: in systems with a larger number of caffeine molecules, the probability of having caffeine molecules with no nearby complexing agent increases, which mechanically lowers the average interaction value—even if a complexing agent is more effective than another one in a less concentrated caffeine solution.

**Fig. 10:**
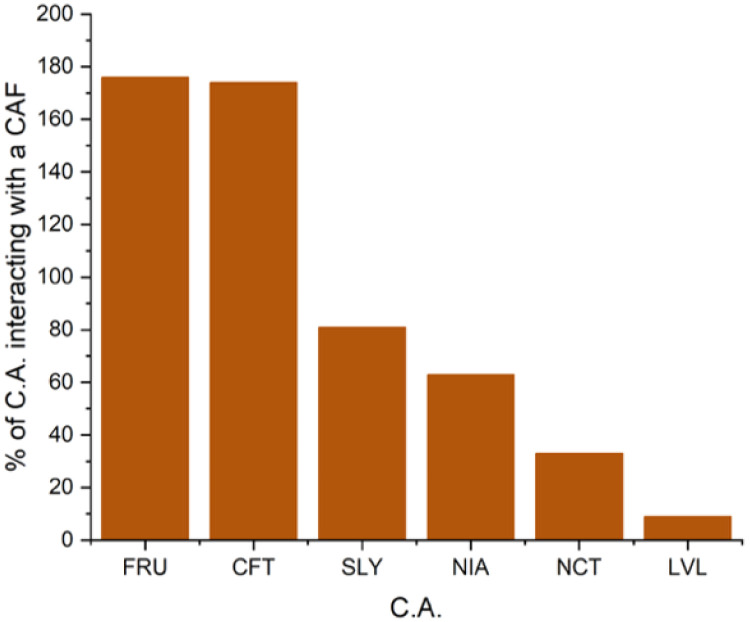
Average number of caffeine molecules interacting with one solubilizing agent. Simulation boxes contain 0.1 mol kg^−1^ of solubilizing agents and caffeine concentration as given in Table 1. See text for details on calculations.

To correct for this bias, data were normalized with respect to the number of caffeine moieties, as follows: (i) a weighted average number of complexing agents per caffeine molecule is computed from the probability distribution of finding 0, 1, 2 or 3 complexing agents, here denoted as P(0), P(1), P(2), P(3), respectively,. This value is then multiplied by the total number of caffeine molecules in the box and finally, this result is divided by the number of complexing agents. This yields an average number of caffeine interactions per complexing agent and allows for a more consistent comparison across systems with different caffeine concentrations as well as a better reflection of the actual efficiency of interactions between a complexing agent and a caffeine moiety. Results are shown in Fig. 10.

With this calculated average number of caffeine moieties interacting with one complexing agent, accounting for caffeine in each box, we observe that sodium ferulate and sodium caffeate are the most efficient at stacking into caffeine fibrils, as the number of caffeine interacting with these molecules is superior to one. Sodium salicylate is also found to interact significantly with caffeine, as almost one molecule of caffeine interacts with each salicylate moiety. Finally, under these new conditions, niacinamide and sodium nicotinate show lowest efficiency reported in this study, with respectively 0.75 and 0.4 molecules of caffeine around each solubilizing agent. These results agree with experimental solubility curves and ROESY experiments.

## Discussion

Hydrotropic solubilization is typically characterized by an exponential increase in solubility beyond a critical hydrotrope concentration, known as the minimum hydrotropic concentration (MHC).^72^ In contrast, the solubility curves obtained in the present study for all investigated methylxanthines display a predominantly linear dependence on the concentration of the solubilizing agent over the explored range. Such linearity is not consistent with a classical hydrotropic mechanism and instead strongly suggests that solubilization is governed by other mechanism. Similar observations have been reported previously. For instance such linear solubility curves were attributed to a complexation mechanism complexation between a cyclodextrin and a solute.^73,74^ Park *et al.* described linear solubility curves for caffeine in the presence of niacinamide, although these systems were still referred to as hydrotropic without discussion of the mechanistic implications of linearity.^44^

Several studies provide a mechanistic basis for complexation-driven solubilization of caffeine in aqueous media. Vranes *et al.* suggested that caffeine solubilization is promoted by π–π stacking interactions with aromatic solubilizers, which prevent caffeine self-aggregation and thereby increase its apparent solubility.^23^ This stacking is favoured when the solubilizer combines an aromatic conjugated system with hydrophilic groups (*e.g.*, carboxylate or hydroxyl) ensuring water solubility of the resulting caffeine–solubilizer complex.

Sherefedin *et al.* further confirmed this hypothesis by demonstrating that π-stacking interactions are the dominant binding mode between caffeine and aromatic acids such as ferulic and synapic acids, consistent with the formation of well-defined 1 : 1 complexes.^75^ Similar π-stacking interactions between caffeine or theophylline and aromatic anions such as sodium benzoate and sodium salicylate were also reported by Aradi *et al.*^76^ Finally, direct experimental evidence for π–π stacking-driven complexation was also recently provided for the caffeine-sodium benzoate system by Shen *et al.* indicating the formation of stable heteromeric complexes and providing structural evidence of π–π stacking between the caffeine and benzoate rings.^77^ Considering these observations, the solubilizing agents investigated in the present work are more appropriately described as complexing agents (CAs) rather than hydrotropes.

All results obtained in this work are in line with the literature. The strong similarity of the solubility curves across methylxanthines, along with their linearity at low concentration, indicates that the same fundamental solubilization processes operate throughout all studied systems. ROESY NMR experiments provide direct evidence of spatial proximity between caffeine and aromatic Cas. Furthermore, the fact that sodium levulinate has no influence on the solubility of caffeine in water clearly demonstrate the role of aromaticity of a complexing agent on the solubilization of methylxanthines.

Moreover, molecular dynamics simulations further confirm that aromatic CAs interact predominantly with caffeine through π–π stacking. Importantly, the hydrophilic substituents of the CAs ensure adequate solvation of a stack, maintaining a hydration shell around the caffeine-CA stacks and preventing precipitation.

The efficiency of a given aromatic CA can be rationalized by considering the specific nature of π–π stacking interactions.

Computational investigations of extended stacking systems demonstrates that dispersion contributions increase rapidly with molecular size and dominate the interaction energy in π–π stacked dimers.^78^ Recent theoretical work further identified molecular polarizability as a key descriptor controlling stacking strength, as dispersion forces originate from instantaneous fluctuations of the electron density and scale directly with electron cloud deformability.^79^ This framework provides a clear explanation for the experimental trend in efficiency observed in this study:Ferulate > caffeate > salicylate > nicotinate > niacinamide

As reported in the SI, the calculated polarizability values of the complexing agents follow the same trend and correlate with solubility enhancements, ROESY NMR observations, and molecular dynamics simulations.

The lower efficiency of pyridine-based CAs (niacinamide and sodium nicotinate) can be understood within the same framework. Substitution of a carbon atom by nitrogen reduces the spatial extent of the π-electron cloud, lowers molecular polarizability, and introduces a permanent dipole. Computationnal analyses of benzene-pyridine and pyridine–pyridine dimers have shown systematically weaker dispersion in pyridine-containing complexes than in benzene dimers.^80^

In aqueous media, the increased polarity and hydrophilicity of pyridine derivatives further favor CA-water interactions over π–π association, thereby reducing complexation efficiency. In contrast, benzene-derived anions, especially methoxy-substituted systems, benefit from higher polarizability and stronger dispersion stabilization within stacked complexes.^81^

Although ROESY NMR and molecular dynamics simulations were not performed for theophylline and theobromine solutions, the similarity of their solubility curves strongly suggests that the same π-stacking-driven complexation mechanism operates. However, structural differences between methylxanthines introduce additional interaction pathways: unlike caffeine, theobromine and theophylline possess N–H group. While π–π stacking remains the dominant interaction driving complexation between methylxanthines and the aromatic complexing agents, as evidenced by the preserved efficiency ranking of the complexing agents across all three methylxanthines, hydrogen bonding appears to provide an additional contribution. This effect is evidenced by the distinct solubility behaviors observed for ferulate, caffeate, and salicylate-based systems when comparing caffeine and theobromine, reflecting the presence of N–H capable of hydrogen bonding in the latter. This is consistent with experimental datas showing caffeine intra and intermolecular interactions to be dominated by π–π stacking, theobromine more by hydrogen bonding, and theophylline to exhibit intermediate behavior.^82^

At higher concentrations, slight inflections or flattening of the solubility curves are observed for the most efficient CAs, notably sodium ferulate and caffeate above 1 mol kg^−1^. These deviations likely reflect the onset of CA self-association (CA-CA). This phenomenon was already reported for sodium salicylate in water by Pawar *et al.* who demonstrated the tendency of such aromatic salts to self-associate.^83^ Beyond a certain concentration, an increasing fraction of CA becomes involve in CA-CA interactions, reducing the concentration of free CA available for interaction with the methylxanthine. In turn, this yield a decrease in the apparent slope of the solubility curve.

Conversely, the limited solubility of sodium ferulate and caffeate significantly enhanced in the presence of caffeine supports a reciprocal complexation process in which caffeine limits CA self-aggregation while aromatic CAs inhibit caffeine self-association, most likely through π–π stacking.

Complexation-driven solubilization through aromatic stacking has been described as an effective strategy for poorly water-soluble compounds by Sanghvi *et al.*^84^ Notably, Ulmann *et al.* reported that sodium ferulate can behave as a hydrotrope for riboflavin, with a clear MHC, emphasizing that the same solubilizer may operate through distinct mechanisms depending on the solute.^32^

Finally, when compared with other solubilization strategies reported in the literature, described in Table 3, aromatic salts such as ferulate, caffeate, and salicylate emerge as among the most efficient approaches for methylxanthine solubilization. In particular, the aqueous CA-based systems investigated here achieve solubilities exceeding those reported in dichloromethane (0.452 mol kg^−1^) and in the DES choline chloride-glycerol, by factors of approximately 9 and 2, respectively.^85,86^ While selectivity toward other coffee constituents was not addressed and may still justify chlorinated solvents in specific contexts, the present results highlight the intrinsic efficiency of aromatic complexation.

**Table 3:** Comparison of the different solubilization strategies for caffeine

Solubilizing systems	Relative efficiency compared to water	Ref.
Pure water	1×	—
Cyclodextrins	<1×	87
ATP	<1×	88
Taurine (0.1mol kg^−1^)	1,07×	89
Mono and disacharrides	<1×	90
Dichloromethane	4,5×	85
Ionic liquids (0.01mol kg^−1^)^a^	2×	91
D.E.S. (ChCl–glycerol)	15×	86
Polyphenolates salts	Up to 30×	This work

a Aqueous solution of ionic liquid.

Overall, our findings demonstrate that the aromatic compounds investigated enhance caffeine solubility in water through π–π stacking-driven complexation and therefore behave as complexing agents rather than hydrotropes. This mechanism provides a practical advantage over classical hydrotropic systems: upon dilution, precipitation is not expected, since any dilution of a saturated solution yields caffeine and CA concentrations that remain below the solubility curve. This property is particularly valuable for formulation processes, whether the ingredient is used as a concentrated solution or in products that undergo dilution during use.

## Conclusions

This study provides a systematic investigation of the solubilization of methylxanthines, namely caffeine, theophylline and theobromine in aqueous solutions containing selected natural solubilizing agents. More precisely, two compounds, namely ferulate and caffeate anions, were tested to enhance the solubility of methylxanthines in water and were compared to known solubilizers of caffeine.

Results demonstrate that these compounds significantly enhance the solubility of methylxanthines in water, up to 300 times the solubility value of theophylline in pure water, depending on the solubilizing agent and the solubilized methylxanthines. It turns out that all these compounds act as complexing compounds and not as classical hydrotropes.

The observed solubilization can be rationalized as an equilibrium between caffeine self-association and association with an aromatic complexing agent. Importantly, this mechanistic interpretation is supported by three independent and mutually consistent levels of evidence: solubility trends and the linearity of the solubilization curves, intermolecular contacts detected by 2D ROESY experiments, and molecular dynamics simulations showing intercalation of aromatic anions within caffeine stacks. Together, these observations strongly indicate that aromatic complexation disrupts caffeine self-aggregation and stabilizes mixed stacked assemblies that remain water-compatible due to the ionic/hydrophilic substituents of the complexing agents. Notably, this complexation solubilization mechanism allows methylxanthines themselves to contribute to the solubilization of complexing agents, such as sodium ferulate and sodium caffeate, unlocking a larger range of concentration. This finding suggests an interplay between the solubilizer and the solubilized compound, which could be exploited for the formulation of highly concentrated aqueous solutions. While we provide theoretical explanation on the difference observed between the different complexing agents, a deeper understanding will be explored in a future theoretical work.

Such systems will be further investigated as sustainable extraction media for the recovery of natural products in upcoming studies. The absence of a critical hydrotropic concentration (MHC) in these systems distinguishes them from conventional hydrotropic solubilization, enabling progressive and controlled dilution without the risk of precipitation of the different solutes. Overall, these findings provide a framework for the development of sustainable, bio-based solubilization strategies applicable to food, nutraceutical, and pharmaceutical formulations. By exploring natural polyphenol derivatives as effective solubilizing agents, this work contributes to the advancement of green chemistry approaches for the extraction and formulation of methylxanthines in aqueous media.

## Author contributions

Eliott Hamonou: methodology, investigation, conceptualization, writing – original draft. Serge Antonczak: methodology, formal analysis, writing – review & editing. Ilya G. Shenderovich: methodology, validation, writing – review & editing. Didier Touraud: conceptualization, visualization. Nicolas Papaïconomou: conceptualization, supervision, writing – review & editing. Werner Kunz: conceptualization, writing – review & editing, validation.

## Conflicts of interest

There are no conflicts to declare.

## Supplementary Material

RA-016-D5RA09568A-s001

## Data Availability

The data supporting this article have been included as part of the supplementary information (SI). Supplementary information is available. See DOI: https://doi.org/10.1039/d5ra09568a.

## References

[cit1] Chemat A., Touraud D., Müller R., Kunz W., Fabiano-Tixier A.-S. (2024). Molecules.

[cit2] Zoumas B. L., Kreiser W. R., Martin R. (1980). J. Food Sci..

[cit3] Vraneš M., Borović T. T., Panić J., Bešter-Rogač M., Janković N., Papović S. (2024). Sustain. Chem. Pharm..

[cit4] Jones F. A. (1985). J. R. Soc. Med..

[cit5] Shively C. A., Tarka S. M. (1984). Prog. Clin. Biol. Res..

[cit6] Balentine D. A. , Harbowy M. E. and Graham H. N. T., in. The Plant and its Manufacture; Chemistry and Consumption of the Beverage, CRC Press, 2019

[cit7] Jalal M. A. F., Collin H. A. (1976). New Phytol..

[cit8] Jiang X., Zhang D., Jiang W. (2014). Eur. J. Nutr..

[cit9] Carman A. J., Dacks P. A., Lane R. F., Shineman D. W., Fillit H. M. (2014). J. Nutr. Health Aging.

[cit10] Flaten V., Laurent C., Coelho J. E., Sandau U., Batalha V. L., Burnouf S., Hamdane M., Humez S., Boison D., Lopes L. V., Buée L., Blum D. (2014). Biochem. Soc. Trans..

[cit11] Rebello S. A., Dam R. M. (2013). Curr. Cardiol. Rep..

[cit12] Qi H., Li S. (2014). Geriatr. Gerontol. Int..

[cit13] Herman A., Herman A. P. (2013). Skin Pharmacol. Physiol..

[cit14] Szendzielorz E., Spiewak R. (2025). Molecules.

[cit15] Góngora C. E., Tapias J., Jaramillo J., Medina R., González S., Restrepo T., Casanova H., Benavides P. (2023). Agronomy.

[cit16] Bertolini M., Ramot Y., Gherardini J., Heinen G., Chéret J., Welss T., Giesen M., Funk W., Paus R. (2020). Int. J. Cosmet. Sci..

[cit17] Cvietusa P., Mascali J. J., Negri J., Borish L. (1996). Ann. Allergy Asthma Immunol..

[cit18] Chang Y.-L., Hsu Y.-J., Chen Y., Wang Y.-W., Huang S.-M. (2017). Oncotarget.

[cit19] Martínez-Pinilla E., Oñatibia-Astibia A., Franco R. (2015). The relevance of theobromine for the beneficial effects of cocoa consumption. Front. Pharmacol.

[cit20] Singh M. , Agarwal S., Agarwal M., and Rachana R., in. Benefits of Theobroma Cacao and its Phytocompounds as Cosmeceuticals, Springer Singapore, Singapore, 2020

[cit21] Tavagnacco L., Fonzo S., D'Amico F., Masciovecchio C., Brady J. W., Cesàro A. (2016). Phys. Chem. Chem. Phys..

[cit22] Tavagnacco L., Gerelli Y., Cesàro A., Brady J. W. (2016). J. Phys. Chem. B.

[cit23] Vraneš M., Borović T. T., Drid P., Trivić T., Tomaš R., Janković N. (2022). Pharmaceutics.

[cit24] Willson C. (2018). Toxicol Rep.

[cit25] Tewabe Gebeyehu B. (2015). Am. J. Appl. Chem..

[cit26] Santos C. I. A. V., Ribeiro A. C. F., Esteso M. A. (2019). Biomolecules.

[cit27] Mejri M., BenSouissi A., Aroulmoji V., Rogé B. (2009). Spectrochim. Acta Mol. Biomol. Spectrosc..

[cit28] Prabu S., Swaminathan M., Sivakumar K., Rajamohan R. P. (2015). J. Mol. Struct..

[cit29] Hervø-Hansen S., Polák J., Tomandlová M., Dzubiella J., Heyda J., Lund M. (2023). J. Phys. Chem. B.

[cit30] Reddy V., Saharay M. (2019). J. Phys. Chem. B.

[cit31] Loukri A., Sarafera C., Goula A. M., Gardikis K., Mourtzinos I. (2022). Appl. Food Res..

[cit32] Ulmann N., Hioe J., Touraud D., Müller E., Horinek D., Kunz W. (2025). J. Mol. Liq..

[cit33] Kabir-ud D., Parveen N., Naqvi A. Z. (2009). Hydrotropic Behavior of Sodium Salicylate in Presence of Additives. J. Dispersion Sci. Technol..

[cit34] Pranata N., of F. G. R., Zakariyya R. C. (2021). Pelita Perkebun..

[cit35] Macalister C. J., Bradshaw T. R. (1903). Lancet.

[cit36] Merinville E., Byrne A. J., Rawlings A. V., Muggleton A. J., Laloeuf A. C. (2010). J. Cosmet. Dermatol..

[cit37] Amann R., Peskar B. A. (2002). Eur. J. Pharmacol..

[cit38] Arif T. (2015). Salicylic acid as a peeling agent: a comprehensive review. Clin. Cosmet. Invest. Dermatol..

[cit39] Chyka P. A., Erdman A. R., Christianson G., Wax P. M., Booze L. L., Manoguerra A. S., Martin Caravati E., Nelson L. S., Olson K. R., Cobaugh D. J., Scharman E. J., Woolf A. D., Troutman W. G. (2007). Clin. Toxicol..

[cit40] Runde T. J. and Nappe T. M., Salicylates Toxicity, StatPearls, StatPearls Publishing, 2023 29763054

[cit41] Madan R. K., Levitt J. (2014). J. Am. Acad. Dermatol..

[cit42] Jeszka-Skowron M., Frankowski R. (2020). LWT--Food Sci. Technol..

[cit43] Lang R., Yagar E. F., Eggers R., Hofmann T. (2008). J. Agric. Food Chem..

[cit44] Park S. I., Lee K. W., Park S. (2022). et al., Aqueous solubility of high concentrated caffeine using hydrotrope and the application to the anti-cellulite cosmetics. Int. J. Adv. Appl. Sci..

[cit45] Román-Montalvo D., Sánchez A., Lorenzana-Licea E., Domínguez Z., Matus M. H. (2024). J. Mol. Liq..

[cit46] Madaan P., Sikka P., Malik D. S. (2021). Recent Pat. Antiinfect. Drug Discov..

[cit47] Gasperi V., Sibilano M., Savini I., Catani M. V. (2019). Int. J. Mol. Sci..

[cit48] Liu S., Qiu Y., Gu F., Xu X., Wu S., Jin Z., Wang L., Gao K., Zhu C., Yang X., Jiang Z. (2022). Int. J. Mol. Sci..

[cit49] Schandelmaier S., Briel M., Saccilotto R., Olu K. K., Arpagaus A., Hemkens L. G., Nordmann A. J. (2017). Cochrane Database Syst. Rev..

[cit50] Ilkhani F. (2016). Niacin and Oxidative Stress: A Mini-Review. J. Nutri. Med. Diet. Care..

[cit51] Zhen A. X., Piao M. J., Kang K. A., Fernando P. D. S. M., Kang H. K., Koh Y. S., Yi J. M., Hyun J. W. (2019). Biol. Ther..

[cit52] Dunbar R. L., Gelfand J. M. (2010). J. Clin. Invest..

[cit53] Press E., Yeager L. (1962). Am. J. Public Health Nation's Health.

[cit54] Eun J.-B., Jo M.-Y., Im J.-S. (2014). Korean J. Food Sci. Technol..

[cit55] Pavlíková N. (2022). Int. J. Mol. Sci..

[cit56] Raj N. D., Singh D. (2022). Health Sci. Rev..

[cit57] Espíndola K. M. M., Ferreira R. G., Narvaez L. E. M., Silva Rosario A. C. R., Silva A. H. M., Silva A. G. B., Vieira A. P. O., Monteiro M. C. (2019). Front. Oncol..

[cit58] Srinivasan M., Sudheer A. R., Menon V. P. (2007). J. Clin. Biochem. Nutr..

[cit59] Zduńska K., Dana A., Kolodziejczak A., Rotsztejn H. (2018). Skin Pharmacol. Physiol..

[cit60] Amorati R., Pedulli G. F., Cabrini L., Zambonin L., Landi L. (2006). J. Agric. Food Chem..

[cit61] Sharma M., Mondal D., Sequeira R. A., Talsaniya R. K., Maru D. A., Moradiya K., Prasad K. (2021). J. Indian Chem. Soc..

[cit62] Sintra T. E., Luís A., Rocha S. N., Lobo Ferreira A. I. M. C., Gonçalves F., Santos L. M. N. B. F., Neves B. M., Freire M. G., Ventura S. P. M., Coutinho J. A. P. (2015). ACS Sustain. Chem. Eng..

[cit63] Shenderovich I. (2019). Molecules.

[cit64] Case D. A. , Ben-Shalom I. Y., Brozell S. R., Cerutti D. S., Cheatham III V. W. D. C. T. E., Darden T. A., Duke R. E., Ghoreishi D., Gilson M. K., Gohlke H., Goetz A. W., Greene D., Harris R., Homeyer N., Huang Y., Izadi S., Kovalenko A., Kurtzman T., Lee T. S., LeGrand S., Li P., Lin C., Liu J., Luchko T., Luo R., Mermelstein D. J., Merz K. M., Miao Y., Monard G., Nguyen C., Nguyen H., Omelyan I., Onufriev A., Pan F., Qi R., Roe D. R., Roitberg A., Sagui C., Schott-Verdugo S., Shen J., Simmerling C. L., Smith J., SalomonFerrer R., Swails J., Walker R. C., Wang J., Wei H., Wolf R. M., Wu X., Xiao L., York D. M. and Kollman P. A., AMBER 2018, University of California, San Francisco, 2018

[cit65] Martínez L., Andrade R., Birgin E. G., Martínez J. M. (2009). J. Comput. Chem..

[cit66] Frisch M. J. , Trucks G. W., Schlegel H. B., Scuseria G. E., Robb M. A., Cheeseman J. R., Scalmani G., Barone V., Petersson G. A., Nakatsuji H., Li X., Caricato M., Marenich A. V., Bloino J., Janesko B. G., Gomperts R., Mennucci B., Hratchian H. P., Ortiz J. V., Izmaylov A. F., Sonnenberg J. L., Williams-Young D., Ding F., Lipparini F., Egidi F., Goings J., Peng B., Petrone A., Henderson T., Ranasinghe D., Zakrzewski V. G., Gao J., Rega N., Zheng G., Liang W., Hada M., Ehara M., Toyota K., Fukuda R., Hasegawa J., Ishida M., Nakajima T., Honda Y., Kitao O., Nakai H., Vreven T., Throssell K., Montgomery Jr J. A., Ogliaro F., Bearpark M. J., Heyd J. J., Brothers E. N., Kudin K. N., Staroverov V. N., Keith T. A., Kobayashi R., Normand J., Raghavachari K., Rendell A. P., Burant J. C., Iyengar S. S., Tomasi J., Cossi M., Millam J. M., Klene M., Adamo C., Cammi R., Ochterski J. W., Martin R. L., Morokuma K., Farkas O., Foresman J. B. and Fox D. J., Gaussian 16, Revision C.01, Gaussian, Inc., Wallingford CT, 2016

[cit67] Bayly C. I., Cieplak P., Cornell W., Kollman P. A. (1993). J. Phys. Chem..

[cit68] Vinkx J., Jenisch L. M., Lemmens E., Delcour J. A., Goderis B. (2022). Biomacromolecules.

[cit69] Klein R., Kellermeier M., Drechsler M., Touraud D., Kunz W. (2009). Colloids Surf. A Physicochem. Eng. Asp..

[cit70] Martin N. H., Floyd R. M., Woodcock H. L., Huffman S., Lee C.-K. (2008). J. Mol. Graph. Model..

[cit71] Lin C., Skufca J., Partch R. E. (2020). BMC Chem..

[cit72] Kunz W., Holmberg K., Zemb T. H. C. (2016). Colloid Interface Sci..

[cit73] Saokham P., Muankaew C., Jansook P., Loftsson T. (2018). Molecules.

[cit74] Loftsson T. (2017). Int. J. Pharm..

[cit75] Sherefedin U., Belay A., Gudishe K., Kebede A., Kumela A. G., Asemare S. (2024). J. Fluoresc..

[cit76] Aradi F., Földesi A. (1985). Magn. Reson. Chem..

[cit77] Shen Y., Xiao Y., Edkins R. M., Youngs T. G. A., Hughes T.-L., Tellam J., Edkins K. (2023). Int. J. Pharm..

[cit78] Cabaleiro-Lago E. M., Rodríguez-Otero J. (2018). ACS Omega.

[cit79] Brandon S., Montgomery G., John H. (2025). Substituent and Heteroatom Effects on π–π Interactions: Evidence That Parallel-Displaced π-Stacking is Not Driven by Quadrupolar Electrostatics. J. Am. Chem. Soc..

[cit80] Hohenstein E. G., Sherrill C. D. (2009). J. Phys. Chem. A.

[cit81] Gussoni M., Rui M., Zerbi G. (1998). J. Mol. Struct..

[cit82] Latosińska J. N., Latosińska M., Olejniczak G. A., Seliger J., Žagar V. (2014). J. Chem. Inf. Model..

[cit83] Pawar K., Desai M. A., Parikh J. (2013). Tenside Surfactants Deterg..

[cit84] Sanghvi R., Evans D., Yalkowsky S. H. (2007). Int. J. Pharm..

[cit85] Anvar S., Golmohammad F. (2010). Solubility of caffeine in water, ethyl acetate, ethanol, carbon tetrachloride, methanol, chloroform, dichloromethane, and acetone between 298 and 323 K. Lat. Am. Appl. Res..

[cit86] Jeliński T., Cysewski P. (2022). Int. J. Mol. Sci..

[cit87] Terekhova I. V., Kumeev R. S., Al’per G. A. (2007). Russ. J. Phys. Chem. A.

[cit88] Vraneš M., Panić J., Tot A., Gadžurić S., Podlipnik Č., Bešter-Rogač M. (2020). J. Mol. Liq..

[cit89] Vraneš M., Borović T. T., Podlipnik Č., Bešter-Rogač M. (2025). J. Taiwan Inst. Chem. Eng..

[cit90] Shumilin I., Allolio C., Harries D. (2019). J. Am. Chem. Soc..

[cit91] Júlio A., Antunes C., Mineiro R., Raposo M., Caparica R., Araújo M. E. M., Rosado C., Fonte P., Santos De Almeida T., Biomed J. (2018). Stat. Biopharm. Res..

