# Supplementary material for: Efficient aqueous solubilization of methylxanthines via complexation with natural polyphenolate salts
Source: RSC Adv. 2026 Apr 23;16(22):20316–29. doi: 10.1039/d5ra09568a (PMC13103865; doi:10.1039/d5ra09568a)
Supplement: RA-016-D5RA09568A-s001 [file RA-016-D5RA09568A-s001.pdf]

Supplementary Information (SI) for RSC Advances.
This journal is © The Royal Society of Chemistry 2026

Supplementary Materials.

E. Hamonou[1,2], S. Antonczak[2], I. G. Shenderovich[1], D. Touraud[1], W. Kunz[1], N. Papaiconomou[2, *]

1: Universität Regensburg, Institute of physical and theoretical chemistry, Regensburg, Germany

2 : Université Côte d'Azur, CNRS, Institut de Chimie de Nice, UMR 7272, Nice, France.

*: To whom correspondence should be addressed: Nicolas.papaiconomou@univ-cotedazur.fr

1H NMR of caffeine and complexing agent aqueous solutions:

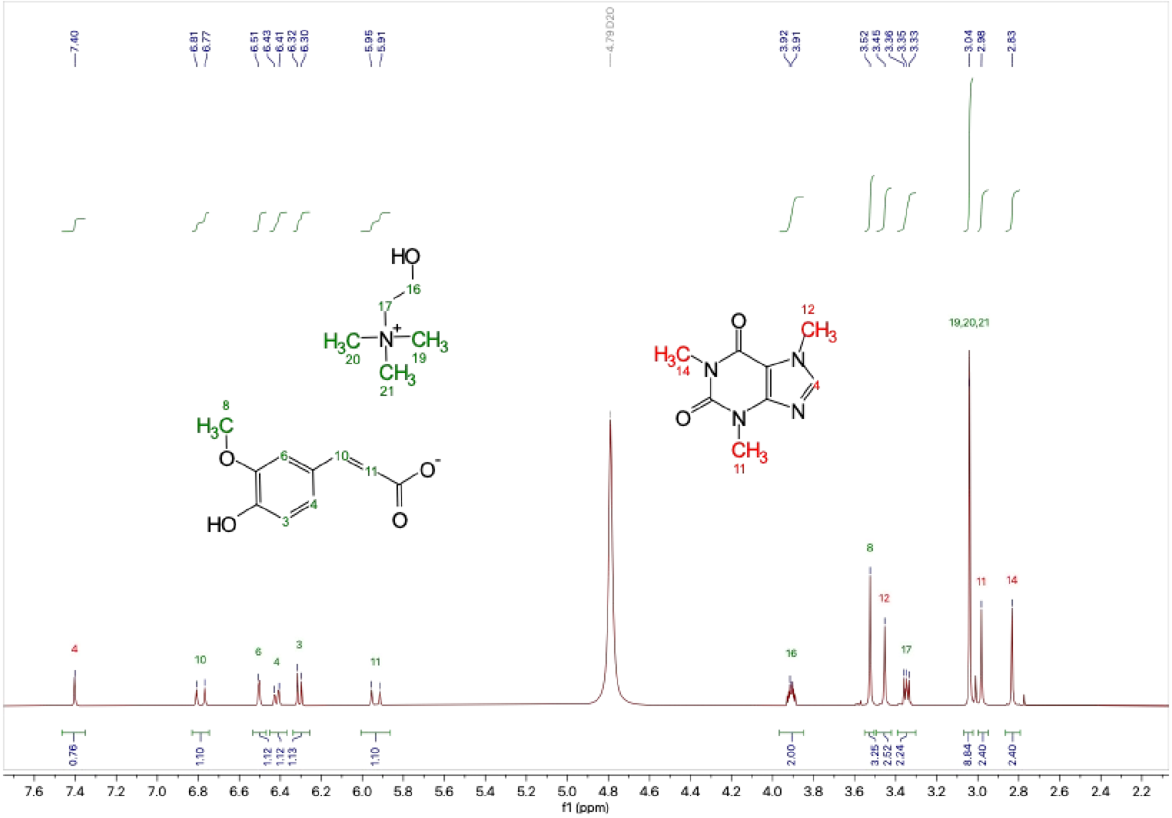

Figure S1: 1H NMR Spectrum of a 3M Cholinium Ferulate and 2.5 M caffeine aqueous solution.

δ_H (400 MHz, D_2O) 7.40 (1 H, s), 6.79 (1 H, d, *J* 16.0), 6.51 (1 H, s), 6.42 (1 H, d, *J* 10.2), 6.31 (1 H, d, *J* 8.1), 5.93 (1 H, d, *J* 16.0), 3.91 (2 H, s), 3.52 (3 H, s), 3.45 (3 H, s), 3.40 – 3.30 (2 H, m), 3.04 (9 H, s), 2.98 (3 H, s), 2.83 (3 H, s).

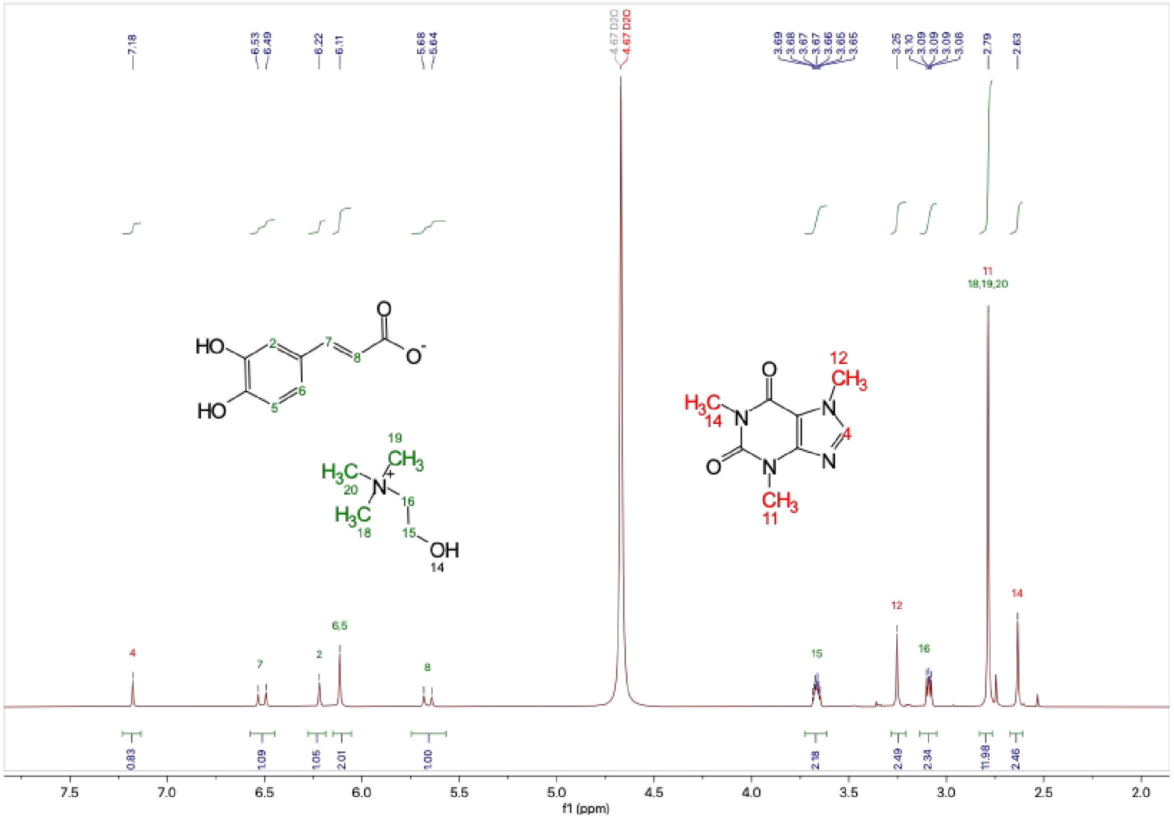

Figure S2: 1H NMR Spectrum of a 3.5M Cholinium Caffeate and 2.5M caffeine aqueous solution

δ_H (400 MHz, D_2O) 7.18 (1 H, s), 6.51 (1 H, d, J 15.9), 6.48 (1 H, s), 6.22 (1 H, s), 6.11 (2 H, s), 5.66 (1 H, d, J 15.9), 3.71 – 3.63 (2 H, m), 3.25 (3 H, s), 3.12 – 3.05 (2 H, m), 2.79 (12 H, s), 2.63 (3 H, s).

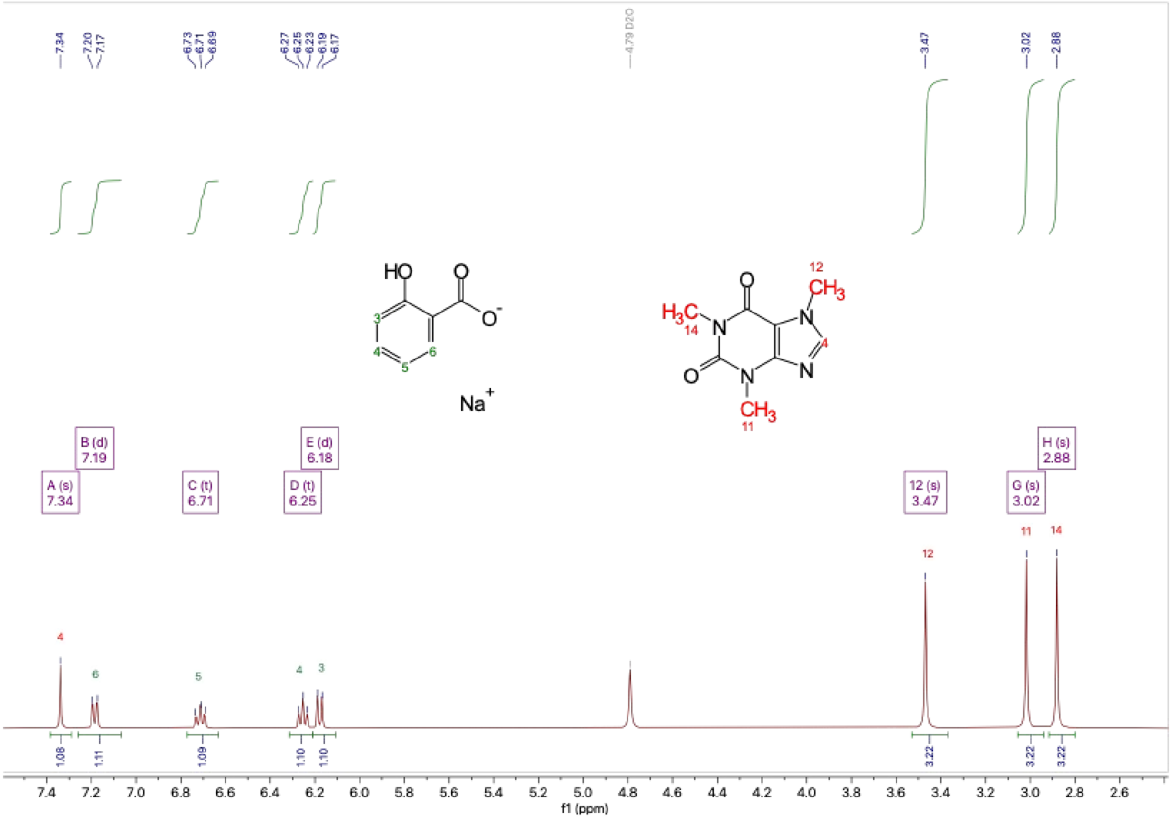

Figure S3: 1H NMR Spectrum of a 3M NaSalicylate and 3M caffeine aqueous solution.

δ $_H$ (400 MHz, D_2O) 7.34 (1 H, s), 7.19 (1 H, d, J 9.5), 6.71 (1 H, t), 6.25 (1 H, t, J 8.1), 6.18 (1 H, d, J 8.2), 3.47 (3 H, s), 3.02 (3 H, s), 2.88 (3 H, s).

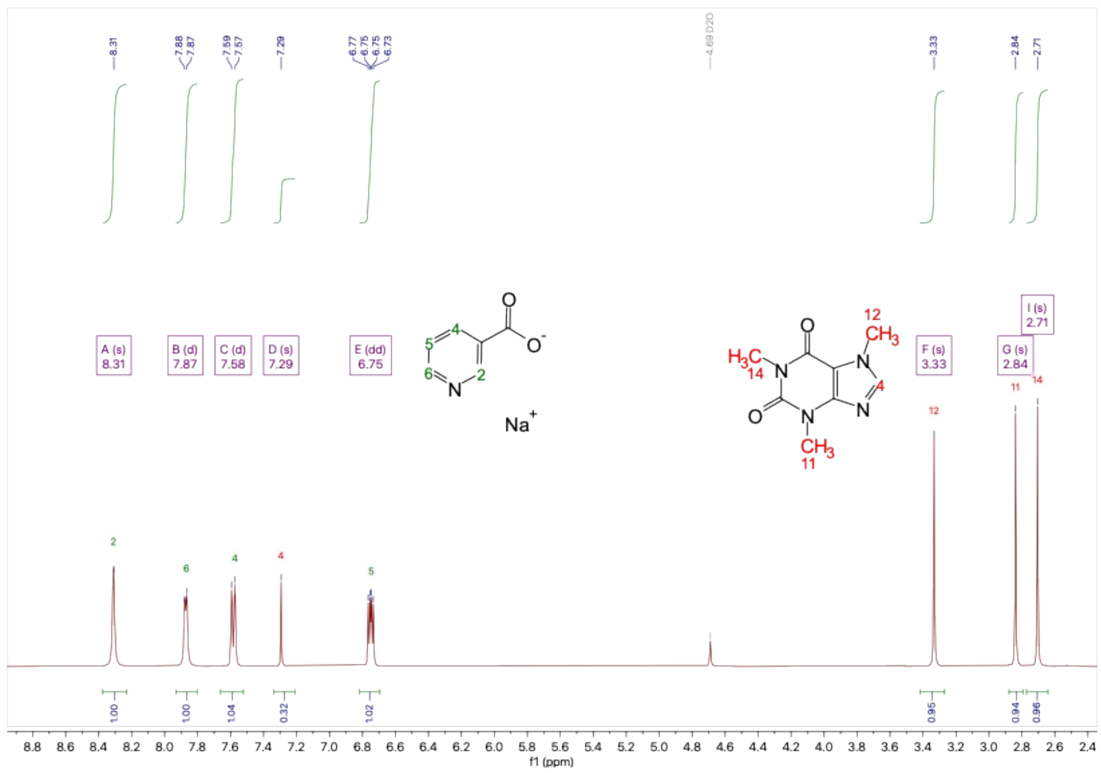

Figure S4: 1H NMR Spectrum of a 3M NaNicotinate 0.8M caffeine aqueous solution.
δ_H (400 MHz, D_2O) 8.31 (1 H, s), 7.87 (1 H, d, J 5.0), 7.58 (1 H, d, J 8.0), 7.29 (1 H, s), 6.75 (2 H, dd, J 7.9, 4.9), 3.33 (3 H, s), 2.84 (3 H, s), 2.71 (3 H, s).

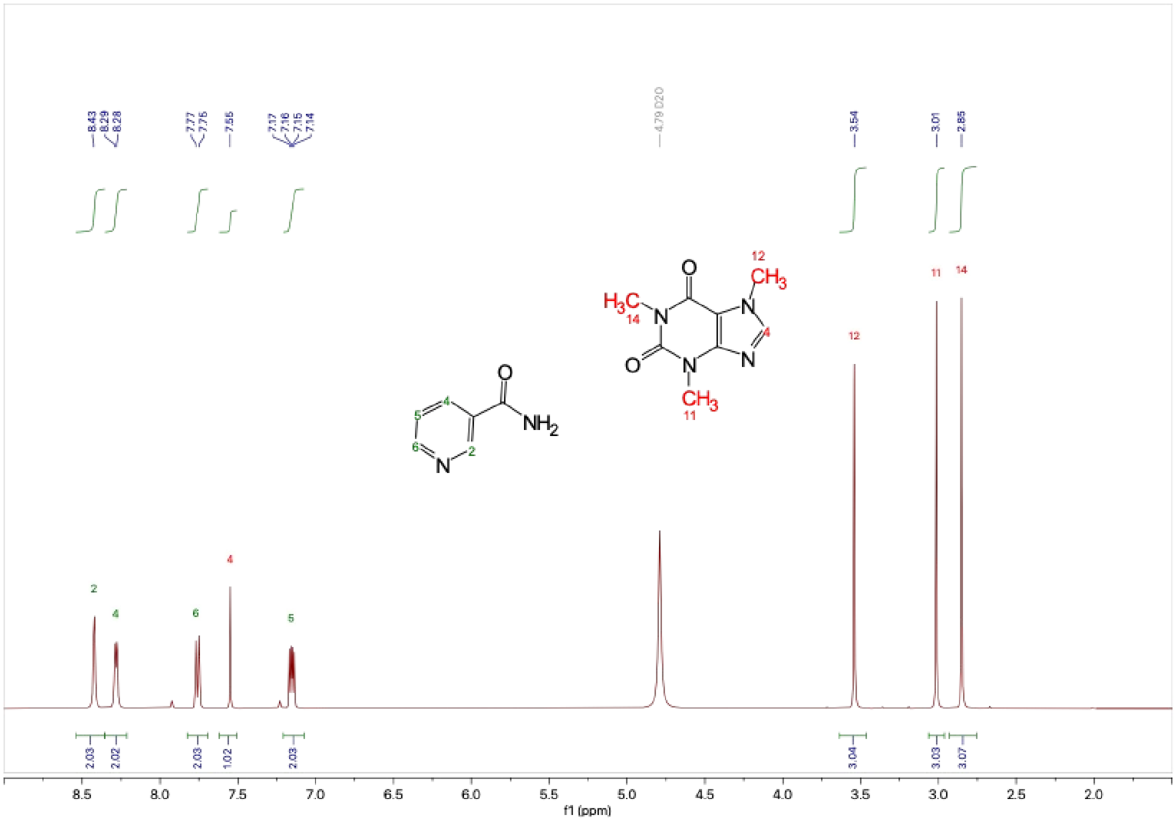

Figure S5: 1H NMR Spectrum of a 3M Niacinamide and 1.5M caffeine aqueous solution.

δ_H (400 MHz, D_2O) 8.43 (1 H, s), 8.29 (1 H, d, J 5.0), 7.76 (1 H, d, J 8.2), 7.55 (1 H, s), 7.15 (2 H, dd, J 8.1, 4.9), 3.54 (3 H, s), 3.01 (3 H, s), 2.85 (3 H, s).

1H NMR shielding due to complexation:

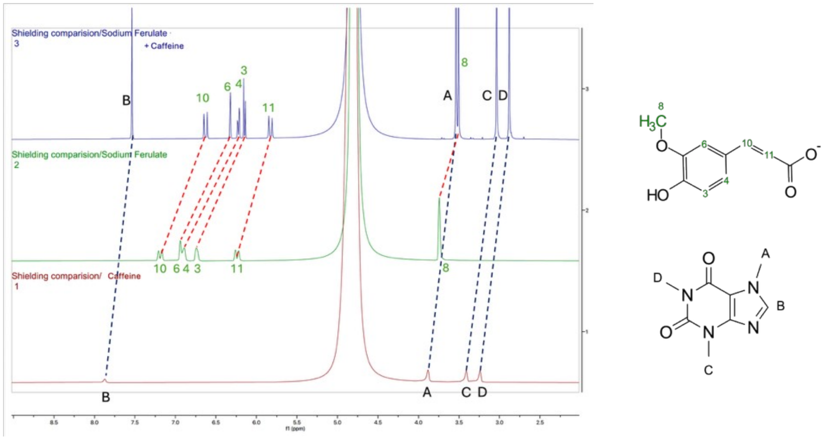

1H I.D.	δ(ppm) of pure component	δ(ppm) for a mixture of caffein and Sodium Ferulate	Δδ
3	6,75	6,15	0,6
4	6,89	6,23	0,66
6	6,94	6,32	0,62
8	3,74	3,50	0,24
10	7,18	6,62	0,56
11	6,24	5,83	0,41
A	3,89	3,53	0,36
B	7,80	7,54	0,26
C	3,42	3,04	0,38
D	3,24	2,88	0,36

Figure S6: 1H NMR peak shifting in aqueous solution of 1) caffein, 2) Sodium Ferulate, 3) Mixture of caffein and Sodium Ferulate ICI RAJOUTER ASSIGNMENTS SUR SPECTRE.

ROESY spectrum analyses for qualitative analysis

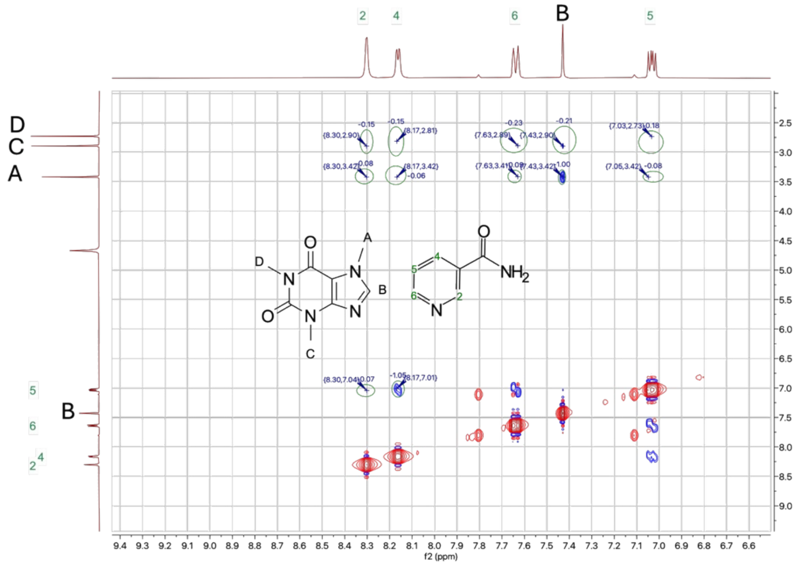

N°	f2 (ppm)	f1 (ppm)	Normalized
1	8.16	7.02	-1.05
2	7.43	3.42	-1.00
3	7.65	2.80	-0.23
4	7.42	2.79	-0.21
5	7.04	2.83	-0.18
6	8.17	2.82	-0.15
7	8.30	2.82	-0.15
8	7.64	3.41	-0.09
9	8.31	3.41	-0.08
10	7.03	3.42	-0.08
11	8.31	7.04	-0.07
12	8.17	3.39	-0.06

Figure S7: ROESY Spectrum of an aqueous solution at 3mol.kg^{-1} of Niacinamide and 1.5mol.kg^{-1} of Caffein

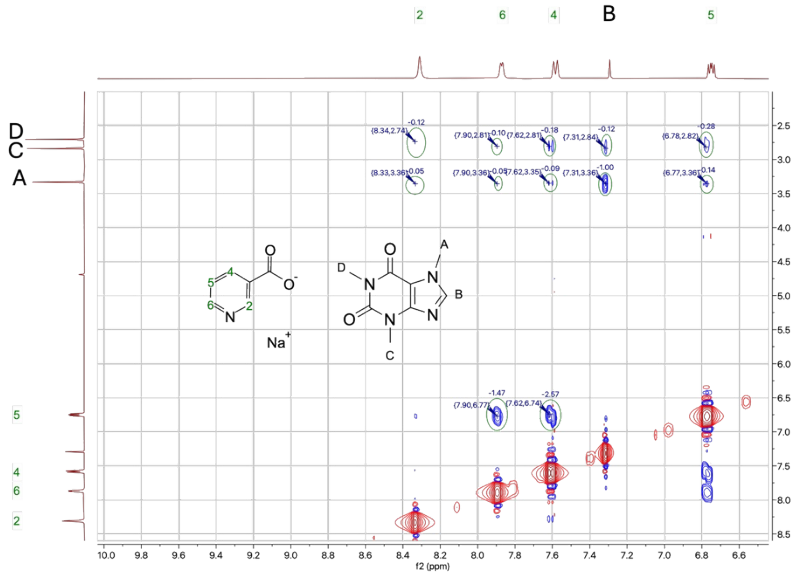

N°	f2 (ppm)	f1 (ppm)	Normalized
1	7.61	6.75	-2.57
2	7.90	6.74	-1.47
3	7.32	3.36	-1.00
4	6.78	2.78	-0.28
5	7.61	2.80	-0.18
6	6.77	3.36	-0.14
7	7.31	2.79	-0.12
8	8.33	2.75	-0.12
9	7.90	2.81	-0.10
10	7.61	3.34	-0.09
11	7.89	3.35	-0.05
12	8.34	3.38	-0.05

Figure S8: ROESY Spectrum of an aqueous solution at 3mol.kg^{-1} of NaNicotinate and 0.9mol.kg^{-1} of Caffein

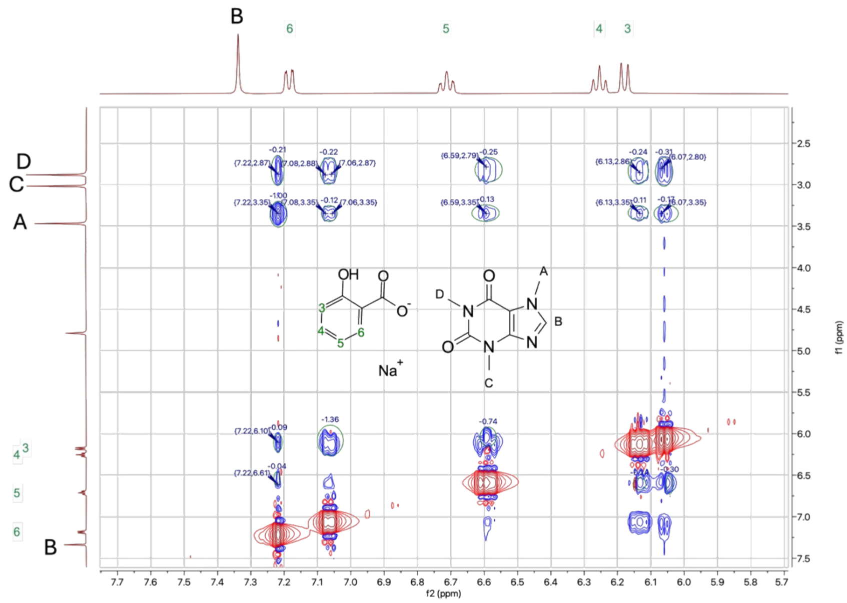

N°	f2 (ppm)	f1 (ppm)	Normalized
1	7.06	6.08	-1.36
2	7.22	3.36	-1.00
3	6.59	6.04	-0.74
4	6.06	2.85	-0.31
5	6.04	6.61	-0.30
6	6.59	2.82	-0.25
7	6.14	2.83	-0.24
8	6.13	6.61	-0.24
9	7.07	2.83	-0.22
10	7.22	2.83	-0.21
11	6.05	3.35	-0.17
12	6.60	3.34	-0.13
13	7.06	3.35	-0.12
14	6.14	3.34	-0.11
15	7.22	6.10	-0.09
16	7.22	6.55	-0.04

Figure S9: ROESY Spectrum of an aqueous solution at 3mol.kg^{-1} of NaSalicylate and 3mol.kg^{-1} of Caffein

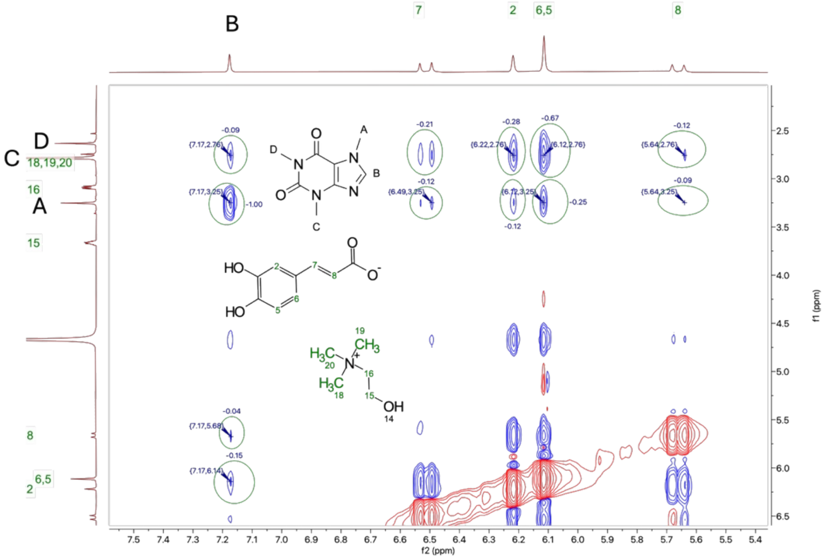

N°	f2 (ppm)	f1 (ppm)	Normalized
1	7.19	3.26	-1.00
2	6.09	2.71	-0.67
3	6.22	2.71	-0.28
4	6.09	3.24	-0.25
5	6.52	2.73	-0.21
6	7.16	6.15	-0.15
7	6.22	3.22	-0.12
8	5.65	2.69	-0.12
9	6.51	3.25	-0.12
10	5.64	3.23	-0.09
11	7.17	2.75	-0.09
12	7.17	5.63	-0.04

Figure S10: ROESY Spectrum of an aqueous solution at 3.5mol.kg^{-1} of ChCaffeate and 2.5mol.kg^{-1} of Caffein

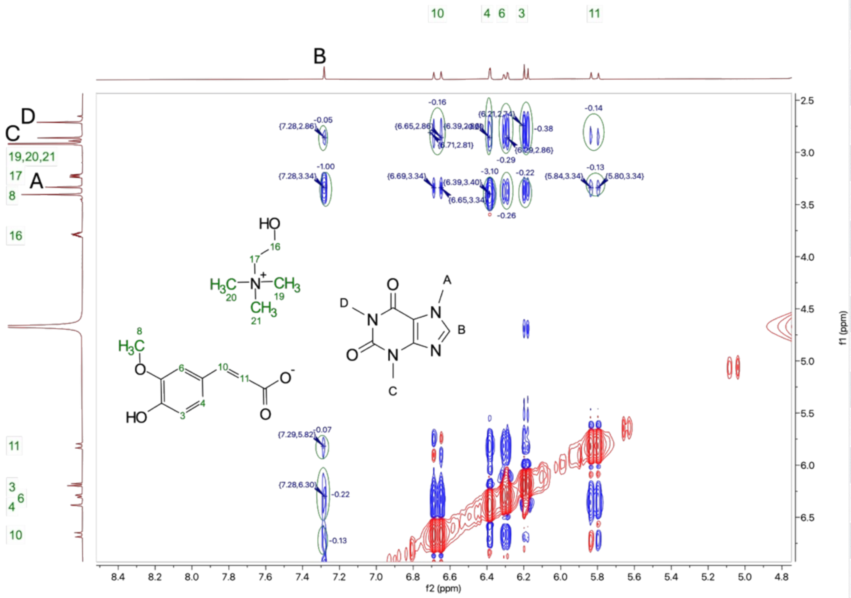

N°	f2 (ppm)	f1 (ppm)	Normalized
1	6.38	3.40	-3.10
2	6.38	5.79	-2.11
3	6.30	5.79	-1.13
4	7.27	3.35	-1.00
5	6.19	2.77	-0.38
6	6.30	2.77	-0.29
7	6.29	3.37	-0.26
8	7.29	6.28	-0.22
9	6.19	3.39	-0.22
10	6.39	2.75	-0.20
11	6.67	2.80	-0.16
12	5.82	2.81	-0.14
13	7.29	6.72	-0.13
14	6.69	5.77	-0.12
15	7.29	5.83	-0.07
16	7.29	2.86	-0.05

Figure S11: ROESY Spectrum of an aqueous solution at 3.5mol.kg^{-1} of ChFerulate and 2.5mol.kg^{-1} of Caffein

Molecular Dynamics snapshots

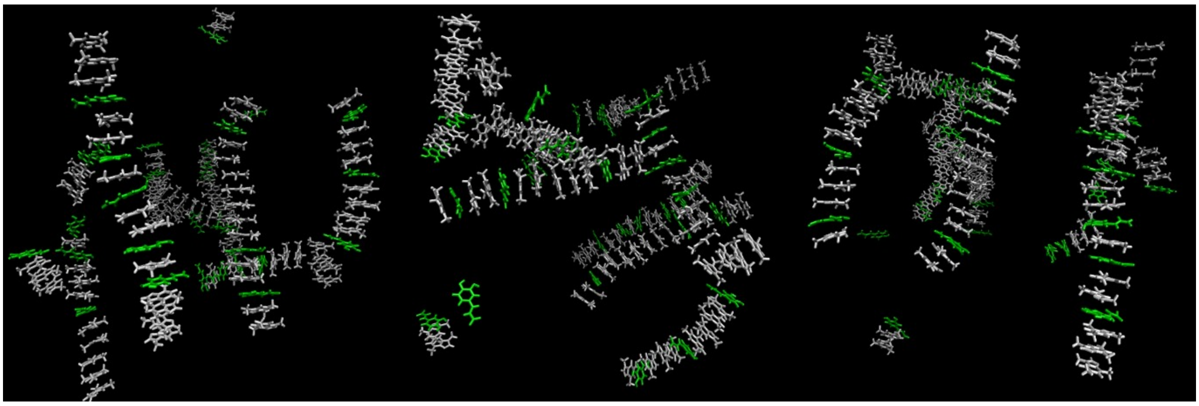

Figure S12: Snapshots from different point of view of the last frame of a 300ns molecular dynamics simulation of a 0.1mol/kg NaCaffeate (Green) and 0.25mol/kg Caffein (white) aqueous solution

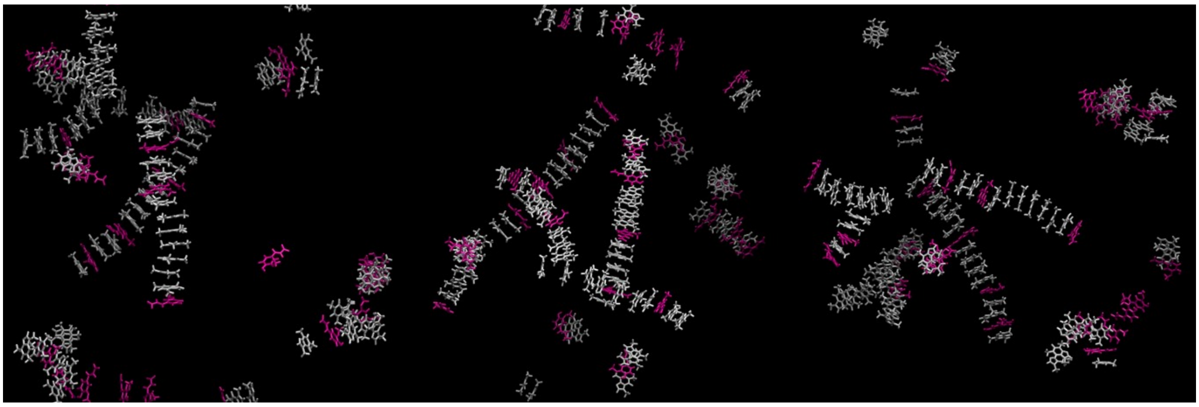

Figure S13: Snapshots from different point of view of the last frame of a 300ns molecular dynamics simulation of a 0.1mol/kg NaFerulate (Pink) and 0.25mol/kg Caffein (white) aqueous solution

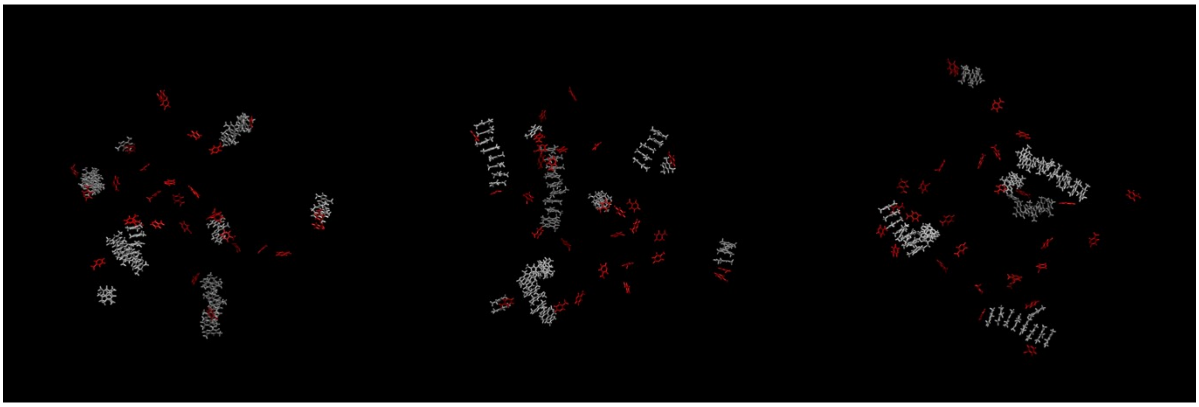

Figure S14: Snapshots from different point of view of the last frame of a 300ns molecular dynamics simulation of a 0.1mol/kg NaNicotinate (Red) and 0.15mol/kg Caffein (white) aqueous solution

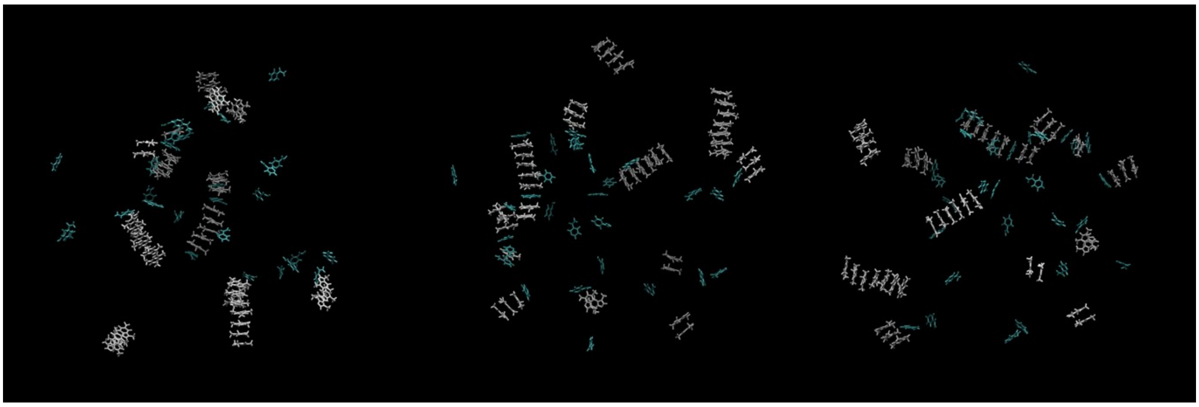

Figure S15: Snapshots from different point of view of the last frame of a 300ns molecular dynamics simulation of a 0.1mol/kg Niacinamide (Cyan) and 0.15mol/kg Caffein (white) aqueous solution

Calculated polarizability of compounds:

Compound	Polarizability (Å^3)
Salicylate	14.9
Niacinamide	12.6

Nicotinate	13.3
Ferulate	23.1
Caffeate	21
Caffeine	18.7
